# A mixed-method investigation of the root causes of construction project delays in Afghanistan

**DOI:** 10.1016/j.heliyon.2025.e41923

**Published:** 2025-01-13

**Authors:** Mohammad Basheer Ahmadzai, Kunhui Ye

**Affiliations:** a School of Management Science and Real Estate, Chongqing University, 83# Shabei Street, Shapingba District, Chongqing, 400045, China; b Faculty of Engineering, Shaikh Zayed University Khost, Khost Province, Afghanistan; c School of Management Science and Real Estate, Chongqing University, 83# Shabei Street, Shapingba District, Chongqing, 400045, China

**Keywords:** Afghanistan, Construction project delays, Conflict-affected countries, Root causes, Security threats

## Abstract

Construction projects in Afghanistan are plagued by significant delays, hindering development efforts and incurring substantial economic losses. Currently, a staggering 79 % of projects experience delays, contributing to a critical infrastructure gap in the nation. This research delves into the root causes of these delays, examining their impact on project outcomes and identifying potential mitigation strategies. By considering the perspectives of five key stakeholder groups—clients, consultants, contractors, project managers, and lecturers—the study aims to develop a comprehensive understanding of the causes contributing to delays. A comparative analysis with other conflict-affected countries, such as Ukraine, Pakistan, Palestine, and Iraq, reveals both shared and unique challenges that hinder construction projects in these regions. The research findings highlight the significant impact of security challenges, corruption, and logistical hurdles on construction project delays in Afghanistan. Tailored strategies are essential to address these multifaceted issues and improve project outcomes. This study offers valuable insights for policymakers, practitioners, and researchers seeking to bolster the performance of Afghanistan's construction sector. A key contribution of this research is its novel focus on security-related causes of construction project delays. By explicitly examining and ranking these causes, the study advances our understanding of how security challenges influence project timelines, a previously underexplored area. Additionally, the comparison with other conflict-affected regions lays the groundwork for broader comparisons and the identification of global best practices. Construction project delays in Afghanistan are influenced by a complex interplay of causes, including security challenges, corruption, and logistical hurdles. Tailored strategies are essential to address these multifaceted issues and improve project outcomes. This research provides valuable insights for policymakers, practitioners, and researchers seeking to bolster the performance of Afghanistan's construction sector.

## 1 Introduction

Construction projects serve as the engines of progress, shaping societies and driving economic development [[1], [2], [3], [4], [5], [6]]. In Afghanistan, however, these projects are plagued by frequent delays that hinder the nation's recovery and growth [[7], [8], [9], [10], [11], [12]]. This study delves into the root causes of construction project delays in Afghanistan, aiming to provide a comprehensive understanding of the complex delay cause contributing to these setbacks. The construction industry in Afghanistan plays a vital role in rebuilding the nation's infrastructure and fostering economic growth [[13], [14], [15]]. Despite its potential, the sector faces numerous challenges that hinder project execution [[16], [17], [18]]. These challenges include high levels of insecurity, complicated contractual parameters, corruption, and inefficient management practices [[19], [20], [21], [22], [23], [24], [25]]. Understanding the multifaceted nature of these delays is essential for developing effective mitigation strategies.

This study focuses on the significant impact of construction project delays on Afghanistan's development. Prolonged delays not only lead to inflated costs and missed opportunities but also divert much-needed resources away from other critical initiatives [[26], [27], [28], [29], [30]]. The delays can have far-reaching consequences, affecting economic growth, social development, and the overall well-being of the Afghan people [31]. Through a mixed-method approach, integrating quantitative analysis and qualitative insights from interviews, this research seeks to investigate the root causes of construction project delays in Afghanistan. By quantifying the risk share of different categories contributing to these delays, the study will highlight root security-related delays and explore the fifteen most impactful causes, categorizing them into specific category. Ultimately, this investigation aims to provide stakeholders—ranging from policymakers to contractors—with actionable insights that inform project management practices, foster collaboration, and drive improvements in Afghanistan's construction sector toward sustained economic growth.

To systematically investigate the factors contributing to construction delays in Afghanistan, this paper is structured as follows: Section 2 provides a comprehensive review of the literature on construction project delays, focusing on global and conflict-affected regions, with an emphasis on Afghanistan. Section 3 discusses the methodology and materials, detailing the research design, including open-ended interviews, questionnaire development, sample selection, quantitative data collection, and the data cleansing process to ensure reliability and validity. Section 4 presents the data analysis, which includes the ranking of delay causes, an in-depth analysis of root causes using Risk Value (RV), Detectability Index (DI), and Risk Priority Number (RPN), and a comparative analysis of key delay causes in Afghanistan and other conflict-affected countries. Section 5 covers the results and discussion, offering insights into the findings and their implications. Finally, Section 6 concludes the paper with a summary of the key findings and actionable recommendations for mitigating construction project delays.

## 2 Literature review

The construction industry in Afghanistan serves as a fundamental pillar in the nation's quest for recovery and economic growth in the aftermath of decades of conflict [[32], [33], [34]]. This sector is not only responsible for developing essential infrastructure but also plays a critical role in rebuilding lives and fostering hope among the Afghan people. Projects like the Turkmenistan–Afghanistan–Pakistan–India (TAPI) Gas Pipeline showcase the industry's potential to revitalize the economy, with expectations of creating thousands of jobs and positioning Afghanistan as a key energy corridor in the region [[33], [34], [35]]. Despite these promising developments, the Afghan construction landscape is fraught with challenges that severely hinder project execution [36]. High levels of insecurity, complicated contractual parameters, rampant corruption, and inefficient management practices contribute to pervasive project delays, which have significant economic and social implications [[37], [38], [39]]. As a nuanced exploration into the delays in Afghanistan's construction projects reveals, while many delays are often attributed to formidable security threats, various intertwined causes demand a comprehensive analysis, including political instability and logistical challenges [33].

Research indicates that a staggering 79 % of construction projects in Afghanistan experience delays, frequently extending well beyond their intended timelines [32]. Such delays have profound consequences that extend to major infrastructure projects, including roads, schools, hospitals, and housing [35]. Each of these plays an essential role in national growth and stability. Among the most significant contributors to these delays, security threats from insurgent groups perpetuate an unpredictable environment that disrupts project plans, inflates costs, and leads to project suspensions [24]. Studies have highlighted that the continuous conflict poses challenges not solely in terms of direct violence, but also through the fear of attack that affects workforce stability and investment attractiveness [[40], [41], [42], [43], [44], [45]]. Contractors frequently incur added expenses associated with security measures, further complicating project budgets [34]. Furthermore, Afghanistan's complex terrain and underdeveloped infrastructure exacerbate logistical challenges, resulting in material shortages and heightened transportation costs that often lead to further delays in project commencement [32].

In addition to security issues, the construction sector in Afghanistan suffers from a significant shortage of qualified labor, particularly in skilled trades like engineering and project management [46]. These workforce deficiencies consequently diminish project quality and efficiency. The lack of trained personnel is exacerbated by financial constraints, including limited access to formal credit and high-interest rates that leave many local contractors at a disadvantage against larger, international firms. Corruption within government institutions adds another layer of complexity; bureaucratic inefficiencies render permitting processes cumbersome, leading to further slowdowns in project approvals. Such systemic corruption disenfranchises legitimate contractors and pushes potential investors away, thus perpetuating a cycle of poverty and stagnation within the construction sector [[47], [48], [49], [50]]. This complex interplay of labor shortages, financing challenges, and corrupt practices culminates in an environment where project delays are not merely common—they are systemic in nature [[51], [52], [53], [54]].

Beyond these region-specific issues, several general causes of construction project delays are prevalent in Afghanistan and globally. Changes in Design or Scope are a common culprit, where modifications to the project blueprint or work requirements, even seemingly minor ones, disrupt established plans and necessitate adjustments, revisions, and rework [[55], [56], [57], [58], [59]]. This throws timeframes into disarray, pushing deadlines further back. Similarly, Unforeseen Site Conditions such as variations in soil composition, the presence of previously unknown underground infrastructure, or even historical artifacts can necessitate adjustments and delays as plans are adapted to address these unforeseen circumstances [[60], [61], [62], [63], [64]]. Adverse Weather Conditions are another uncontrollable factor, with unpredictable events like storms, floods, or extreme temperatures disrupting progress and forcing delays until conditions improve and work can safely resume [65,66]. Delayed Permits or Approvals also significantly stall project progress. Obtaining necessary permits and approvals from relevant authorities is often a time-consuming process, and any delays here can halt a project's momentum [67]. Additionally, the Unavailability of Labor or Materials acts as a roadblock, with shortages leading to slowdowns or even standstills in project activities. Equipment Failure or Breakdown can also be a disruptive force, with necessary repairs or replacements causing delays while equipment is unavailable [68].

The Gardez-Khost Highway project serves as a poignant illustration of the multifaceted obstacles facing Afghanistan's construction industry. Initially budgeted at $69 million, the project ballooned to a staggering $176 million, primarily due to security-related expenses and allegations of corruption among local power brokers. This project highlights not only the financial strains associated with prolonged timelines but also the direct consequences of inadequate planning and resource allocation [33]. The complex subcontracting arrangements led to confusion and inefficiency, further impeding progress. Ultimately, the highway remains incomplete and unsafe, underscoring the urgent need for effective project management strategies that can mitigate such challenges [31]. The myriad setbacks encapsulated in this project underscore the pressing need to address construction delays as pivotal to Afghanistan's broader development agenda [22]. Prolonged project timelines not only lead to substantial economic losses due to inflated costs but also divert much-needed resources away from other critical developmental initiatives [69].

While existing studies have documented various contributors to construction delays in Afghanistan, they often overlook an in-depth analysis of specific security threats and their nuanced interactions with project variables. Security issues are indeed often named as significant impediments; however, a deeper exploration into the specific types of security challenges—such as the geographic location of projects and the nature of local rivalries—has yet to be fully realized in academic literature [33]. This research seeks to bridge this gap by investigating the multifaceted nature of security-related causes that contribute to delays. By employing a mixed-method approach, the study will analyze both qualitative and quantitative data to explore how these security concerns intersect with other project variables. Additionally, the research will use the Risk Priority Number (RPN) method to evaluate delay causes systematically, emphasizing the importance of understanding these interrelations to develop effective mitigation strategies.

Implications of this research are significant, not just for the construction sector in Afghanistan but also for the academic discourse around construction project delays in developing nations. As stakeholders such as government agencies, businesses, and civil society actors come together to grapple with the challenges inherent in the Afghan context, the findings from this investigation promise to empower them with a clearer understanding of the obstacles they face. By identifying key delay-causing elements and their specific impacts, this study will provide essential insights that stakeholders can use for benchmarking purposes, risk assessment, and preventive action throughout the project lifecycle. Furthermore, the research aims to equip project managers with actionable insights into how best to navigate the unique challenges present in Afghanistan, ultimately improving project outcomes and fostering development in a country in dire need of effective infrastructure solutions.

The significance of understanding construction project delays is underscored by the broader implications for global development efforts [[70], [71], [72], [73], [74]]. The insights gathered from this study will not only be directly applicable to Afghanistan but could also serve as a valuable resource for other developing nations grappling with similar security challenges and construction-related obstacles. By recognizing commonalities in the experiences of countries emerging from conflict or facing similar socio-economic barriers, this research has the potential to contribute to a global dialogue on effective project management in post-conflict settings. In conclusion, by offering a nuanced and comprehensive understanding of the root causes of construction project delays, this study hopes to illuminate pathways for improved practices and collaborative efforts aimed at mitigating the adverse effects of such delays, fostering greater resilience in Afghanistan's construction sector, and, ultimately, enabling the country to leverage its construction industry as a driver of national recovery and growth.

According to the information collected from the literature review, Table 1, Table 2, Table 3 below indicate the list of general causes for construction project delays**.**

**Table 1:** List of the government-related causes of delay with their references.

Delay Causes	Reference	Delay Causes	Reference
Lack of Controlling	[12,15,16,19,22,23,28,29,29,31,34,37,40,42,52,54,63,65].	Certifications of municipality	[12,13,16,21,[23], [24], [25],^29^,^35^,^37^,^38^,^41^,^69^].
Late payments		Permissions for foreign laborers	
Scope changes		Bureaucracy	
Late in approving design		Certification of urban planning office	
Delay in shop drawings		Permission from order of engineers	
Poor communication		Changes in the principles	
Providing site access		Poor government judicial system	
Decision making delays		Changes of design imposed	
Corruption		Giving projects to the lowest offer

**Table 2:** Consultant-related delay causes with their references.

Delay Causes	Reference	Delay Causes	Reference
Contract time is not enough	[9,[14], [15], [16],^18^,[18], [18], [19], [20],^27^,^29^,^42^,^50^,^55^,^59^,^61^,^69^].	Lack experience consultant	[1,4,7], 38] [3,8,9,37,41,47].
Delay in the checkup		Poor project management	
Delay in the approving of changes		Delay in the main changes	
Rigidity of consultant		Complication of the design	
Delay in the reviewing		Unsatisfactory data for design	
Insufficient experience		unskilled design team	
Errors in the design documents		Misunderstanding of requirements	
Poor data collection during survey		Incomplete project design	
Un-use of update software		Unclear details in drawings	
Designer lake experience		Project design get changed	
Unskilled drawings		The consultant's staff not on site	
Mistakes in the contract		Delays in making design documents

**Table 3:** Contractor-related causes of delay with their references.

Delay Causes	Reference	Delay Causes	Reference
Inadequate management	[4,[14], [15], [16],^16^,[23], [24], [25], [26],^29^,^30^,^38^,^39^,^44^,^45^,^51^,^65^,^69^,^71^,^76^,^82^,^86^].	Low productivity level of labors	[1,3,4,6,11,15,15,[19], [20], [21], [22],^27^,^30^,^34^,[39], [40], [41],^46^,^49^,^57^,^61^,^63^,^69^].
Financial problems		Weak motivation	
Delay in site mobilization		Inaccessibility of utilities	
Lack of experience		Effect of community	
Poor contractor's work		Accident during construction	
Rework due to errors		Delay in providing services	
Poor financial control on site		Lake of the contractor experience	
Ineffective project planning		Unsuitable construction techniques	
Little production		Unskilled project team	
Delay because of sub-contractors		Poor site management and regulation	
Insufficient construction methods		Insufficient site investigation	
Poor site organization		Unsuitable contractor's guidelines	
Poor communication		Lack of construction materials	
Unsuccessful planning		Delay in the delivery of materials	
Change of sub-contractors		Damage of sorted material	
Difficulties in the financing		Shortage of labors	
Conflicts of sub-contractor's		Inadequate material storage	
Unavailability of resources		Poor quality	
Low equipment-operator's skill		Changeable suppliers	
Low productivity of equipment		Employment injuries	

## 3 Methodology and materials

**Fig. 1:**
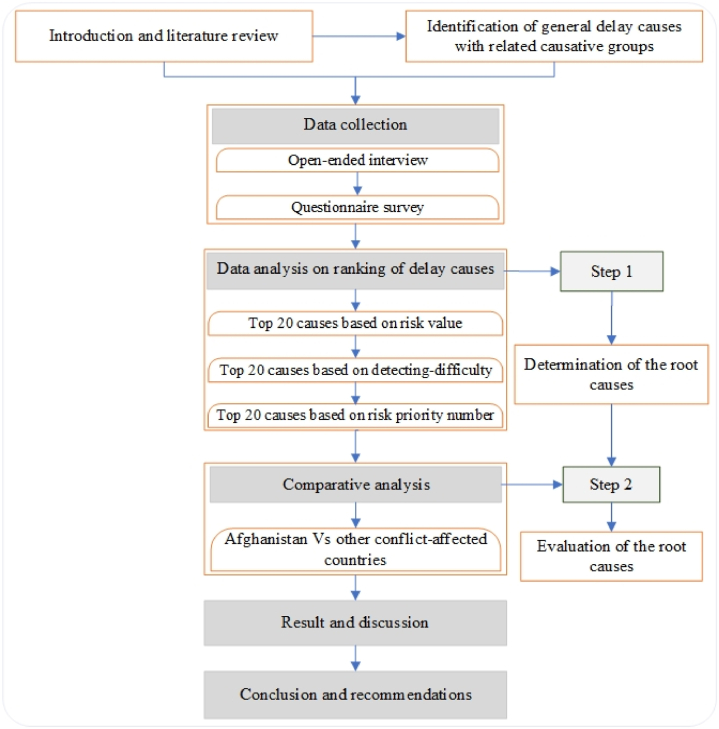
Framework of the research.

Following Fig. 1 is the flow chart for the research.

### 3.1 Open-ended interview

Eleven participants (4 project managers, 4 contractors, 3 consultants) were interviewed for this study, representing a diverse range of experience and expertise within the Afghan construction sector. Project managers provided insights into overall project planning and execution. Their experience ranged from 7 to 13 years, with a mix of educational backgrounds including Masters and PhDs. Contractors offered perspectives on construction practices and challenges. Their experience varied from 5 to 15 years, and they held positions like work director, construction supervisor, and head of construction companies. Finally, consultants contributed knowledge on specific areas like design, quality control, and administration. Their experience spanned 13–17 years, with specializations in structural engineering, quality supervision, and administrative and finance. These interviews aimed to uncover their extent, underlying causes, responsible categories, and to get feedback on participant selection for a forthcoming questionnaire survey. Key questions asked experts to describe a recent delayed project, identify contributing delay causes, pinpoint the most important causative groups, and suggest improvements to the proposed participant categories (project managers, contractors, consultants). In Afghanistan, construction projects rarely finish on time, with a staggering 79–85 % experiencing delays. Interviews with 11 industry experts revealed a web of factors behind these setbacks, ranging from government corruption and late payments to design changes and security threats.

These delays stem from four main categories: government, consultants, contractors, and security issues. Governments often change designs, while inefficient contractors struggle with cash flow and rework. Consultants face challenges like late design approvals and inaccurate surveys. But perhaps the most unique challenge is security: local authorities, tribal conflicts, and even war can disrupt projects. To pinpoint the most critical causes, the research now moves to a questionnaire survey. By expanding the participant pool to include clients, consultants, contractors, project managers, and even lecturers, the study seeks to comprehensively understand and ultimately mitigate the delay causes plaguing Afghan construction projects.

### 3.2 Questionnaire design

Building upon existing research ([49,57,77]), the initial questionnaire concept was further refined through open-ended interviews with Afghan construction experts. This led to the development of the questionnaire for quantitative data collection in the Afghan construction context. The questionnaire is dedicated to evaluating delay causes, categorized into government, consultant, contractor, and security-related issues. The questionnaire commences with an introduction to the study's topic and aims. Participants are then asked to provide their general information, including name, contact details, age, occupation, education, experience, and regional details. Subsequently, participants are prompted to furnish essential project details such as project name, client or funding source, location, and total project value. Following the general information section, the questionnaire proceeds with four ranking sections focused on different categories of delay causes. Participants are requested to rank the severity, frequency of occurrence and detection probability (on a scale of 1–5) for each cause of delays. This structured approach aims to discern root causes of delays in Afghan construction projects.

### 3.3 Selection of sample size

This research uses Hogg and Tanis equations (1), (2) for sample size [75].

(1) $$n=\frac{m}{1+\left(m-1\right)/N}$$

Where: n = limited sample size, m = unlimited sample size, N = available population, as well as m is calculated by the following equation:

(2) $$m=\left\{Z^{2}*P*\left(1-P\right)\right\}/e^{2}$$

Where: Z is value of confidence used 2.575 for 99 %, 1.96 for 95 %, and 1.645 for 90 % levels of confidence, P is the value of the required population, which is suggested 0.5 by Sincich et el [74]. So, following is the required size of sample for this research.

(2∗) $$m={\{\left(1.96\right)}^{2}*0.5*\left(1-0.5\right)\}/{\left(0.05\right)}^{2}=385$$

As the total available stakeholders for the 100 construction projects are 800, then the required numbers of the participants can find with equation. (1) as follow:

(1∗) $$n=\frac{385}{1+\left(385-1\right)/800}=260.135135=260$$

Regarding the above calculation, 260 stakeholders are required for the data collection to represent the total of 800 stakeholders for the research.

### 3.4 Quantitative data collection

Quantitative data was collected through a questionnaire survey using two methods: online and offline data collection. For the online method, the questionnaire was distributed to participants via email, messenger platforms like WhatsApp, and other online channels. Simultaneously, the offline method employed paper-based questionnaires. Participants in this survey included key stakeholders in Afghan construction projects: clients, government representatives, consultants, contractors, lecturers, and project managers. These individuals are directly involved in Afghan construction projects. The characteristics of the participants are presented in Table 4, Table 5, Table 6, Table 7, Table 8. A total of 100 Afghan construction projects (50 urban and 50 rural) were selected for quantitative data collection, ensuring diversity in terms of zone, size, and type. Six main project types were included: educational (21), water supply (16), road (21), irrigation (19), military (7), and hospital (16). The projects were included from 2019 to 2022.

**Table 4:** Participants ages.

Description	Client	Consultant	Contractor	Lecturer	Project manager	Total
20–30 years old	20	23	18	27	30	118
31–40 years old	22	20	25	18	16	101
41–50 years old	11	11	9	14	15	60
Above 50 years old	7	5	7	4	3	26
Total	60	59	59	63	64	305

**Table 5:** Education information of participants.

Description	Client	Consultant	Contractor	Lecturer	Project manager	Total
PhD	8	8	7	15	9	47
Master	26	21	17	29	31	124
Bachelor	25	28	22	19	23	117
High school	1	2	13	0	1	17
Total	60	59	59	63	64	305

**Table 6:** Participants experience.

No	Client	Consultant	Contractor	Lecturer	Project manager	Total
Less than three years	7	9	12	8	13	49
Three to six years	25	23	23	15	17	103
Six to ten years	19	20	14	23	19	95
More than ten years	9	7	10	17	15	58
Total	60	59	59	63	64	305

**Table 7:** Professions of participants.

No	Client	Consultant	Contractor	Lecturer	Project manager	Total
Civil engineer	31	22	24	29	20	126
Construction manager	11	15	19	14	18	77
Site Supervisor	11	13	10	13	17	64
Quantity Surveyor	7	9	6	7	9	38
Total	60	59	59	63	64	305

**Table 8:** Participants provincial details.

Description	Client	Consultant	Contractor	Lecturer	Project manager	Total
Central Highland	9	6	5	7	8	35
Central Region	8	8	10	9	7	42
Eastern Region	7	5	10	4	9	35
North Eastern Region	9	10	11	5	8	43
Northern Region	7	6	4	8	10	35
South Eastern Region	8	10	7	14	11	50
Southern Region	5	7	8	10	6	36
Western Region	7	7	4	6	5	29
Total	60	59	59	63	64	305

### 3.5 Data cleansing, reliability, and validity

Data accuracy and integrity were ensured through a thorough data cleansing process, alongside reliability and validity checks. Before the analysis, the dataset was cleaned by removing unfilled and incomplete questionnaires. Out of 400 distributed questionnaires, 314 were returned, reflecting a high response rate of 78.5 %. After removing nine incomplete responses, 305 valid questionnaires were used for analysis. This careful cleansing process ensured that only high-quality, complete data contributed to the research findings. Reliability was enhanced by conducting a pilot survey to refine the questionnaire and using a mixed-methods approach, combining qualitative interviews with quantitative questionnaires. An anonymous questionnaire design encouraged honest and unbiased responses. The reliability of the questionnaire was assessed using Cronbach's Alpha, which demonstrated strong internal consistency across various respondent categories, with alpha values ranging from 0.742 to 0.874. These scores confirmed the reliability of the data, ensuring consistent findings across different sectors involved in the study [[76], [77], [78], [79], [80]]. To ensure validity, the study engaged key stakeholders in the Afghan construction industry and collected data directly from actual construction projects. In-depth interviews with industry experts provided valuable insights, while a pilot test helped identify potential data collection issues. Additionally, multicollinearity tests confirmed the robustness of the data. The combination of qualitative and quantitative approaches ensured a comprehensive analysis of delay causes in construction projects, providing accurate, relevant, and trustworthy findings for the research.

## 4 Data analysis

### 4.1 Analysis on ranking of delay causes

This research adopts the methodology of Fani Antoniou (2021) to rank construction project delays. It then analyzes the collected data using the Risk Priority Number (RPN) [81]. The Risk Priority Number (RPN) method, originally introduced by Fine and Kinney (1971) for construction safety hazards, offers a valuable framework for prioritizing potential risks in various contexts, and while initially used for safety concerns, its core principles can be effectively applied to identify and prioritize critical issues in construction projects [82]. Ayyub (2012) describes a method for prioritizing risks using a score called the (RPN). This score considers the severity and likelihood of a risk. Higher RPNs indicate greater risk, and these are the issues that demand the most attention in construction projects [83,84]. The Risk Priority Number (RPN) provides a simple yet powerful tool for ranking risks in businesses and construction projects. It achieves this by breaking down severity, occurrence, and detectability into sub-criteria. This structured approach allows for both qualitative judgments and reliable data to be incorporated in the analysis, avoiding the need for imprecise conversion tables [85]. RPN is calculated by multiplying ratings assigned to severity, how often a failure might occur, and the likelihood of detecting such a failure [86]. These causes are then combined into a single RPN score, providing a clear way to prioritize critical delays in construction projects [87]. Following equations (3), (4), (5), (6), (7)) is used to rank the causes of construction project delays.

The risk value (RV), the risk value assesses the overall significance of each delay cause by considering both its frequency and severity, is given by:

(3) RV=SI.FI

It combines the insights from both occurrence and severity to provide a holistic view of which delay causes are most important to address [88,89].

The Severity Index (SI), evaluates the seriousness or impact of each delay cause, is given by:

(4) $$SI=\sum_{i=1}^{5}\left(a_{i}.n_{i}\right)/5N$$

Where a = conveying weightiness, given for every response (ranging from 1 for very low severity to 5 for very high severity), n = frequency responses for every cause of delay, and N = response total amount [88,89].

The Frequency occurrence index (FI), measures the frequency or how often specific delay causes occur during construction projects, is given by:

(5) $$FI=\sum_{i=1}^{5}\left(b_{i}.n_{i}\right)/5N$$

Where b = conveying weightiness, given for every response (ranging from 1 for not happened to 5 for always happened), n = frequency responses for every cause of delay, and N = total number of the response [88,89].

The detectability index (DI), Evaluate the probability of identifying each delay cause before it significantly impacts the project. Higher scores indicate a better chance of early detection, while lower scores suggest potential difficulty in noticing the issue early on. It is calculating by:

(6) $$DI=\sum_{i=1}^{5}\left(c_{i}.n_{i}\right)/5N$$

Where the value c represents the assigned weightiness score for each response, ranging from 1 (very low detectability) to 5 (very high detectability). n refers to the number of responses received for each specific delay cause [88,89]. Achronic RV (Risk Value) and DI (Detectability Index) are two key metrics used in risk assessment and management to quantify and prioritize risks based on their potential impact and likelihood of occurrence. By combining Achronic RV and DI, you can prioritize risks based on their potential impact and detectability, allowing you to focus your mitigation efforts on the most critical issues.

Risk priority number (RPN) was calculated the severity of event (S), the probability of occurrence (F) and probability of detection (D) according to the following formula:

(7) RPN=SI.FI.DI

Based on the data analysis in Table 9, Table 10, Table 11, Table 12, which delve into the severity, frequency, risk value, detectability, and Risk Priority Number (RPN) indices, this section presents the ranked impact of delay causes in construction projects across Afghanistan.

**Table 9:** Ranking of the government-related delay causes.

Delay causes	SI	R	FI	R	RV	R	DI	R	RPN	R
Lack of Controlling	0.4850	17	0.5019	18	0.2434	18	0.5019	16	0.1222	18
Late payments	0.6730	3	0.6840	3	0.4603	3	0.6803	1	0.3132	1
Scope changes	0.5000	16	0.5167	17	0.2584	17	0.5807	7	0.1500	13
Late in approving design	0.6120	6	0.6155	8	0.4301	6	0.6766	2	0.2466	3
Delay in shop drawings	0.5270	8	0.5428	10	0.2860	10	0.6691	3	0.1914	7
Poor communication	0.5040	14	0.5204	15	0.2623	15	0.6082	5	0.1595	11
Providing site access	0.6810	2	0.6914	2	0.4709	2	0.6364	4	0.2997	2
Decision making delays	0.5080	12	0.5242	13	0.2663	13	0.6045	6	0.1610	10
Corruption	0.6850	1	0.6952	1	0.4762	1	0.5093	14	0.2425	4
Certifications of municipality	0.5040	14	0.5204	15	0.2623	15	0.5688	8	0.1492	14
Permissions for foreign laborers	0.5150	11	0.5316	12	0.2738	12	0.5279	10	0.1445	16
Bureaucracy	0.6540	5	0.6654	5	0.4352	5	0.5465	9	0.2378	5
Certification of urban planning office	0.4850	17	0.6193	7	0.3004	9	0.5167	11	0.1552	12
Permission from order of engineers	0.5080	12	0.5242	13	0.2663	13	0.5093	13	0.1356	17
Changes in the principles	0.5690	7	0.5836	9	0.3321	7	0.5056	15	0.1679	8
Poor government judicial system	0.5190	10	0.6394	6	0.3319	8	0.4870	18	0.1616	9
Changes of design imposed	0.5230	9	0.5390	11	0.2819	11	0.5160	12	0.1455	15
Giving projects to the lowest offer	0.6690	4	0.6803	4	0.4551	4	0.4989	17	0.2271	6

Note: SI = severity index, R = Rank, FI = frequency index, RV = risk value, DI = detectability index, RPN = risk priority number.

**Table 10:** Ranking of the consultant related delay causes.

Delay causes	SI	R	FI	R	RV	R	DI	R	RPN	R
Contract time is not enough	0.5643	24	0.4892	24	0.2761	24	0.6885	1	0.1901	18
Delay in the checkup	0.6818	7	0.6186	5	0.4218	5	0.5665	13	0.2389	7
Delay in the approving of changes	0.6424	15	0.5673	13	0.3644	14	0.681	2	0.2482	5
Rigidity of consultant	0.6119	20	0.548	17	0.3353	20	0.5643	14	0.1892	19
Delay in the reviewing	0.7665	1	0.6914	1	0.5300	1	0.6587	3	0.3491	1
Insufficient experience	0.6713	10	0.626	4	0.4203	6	0.5606	15	0.2356	8
Errors in the design documents	0.6097	22	0.5346	21	0.3259	22	0.5509	17	0.1796	22
Poor data collection during survey	0.7442	3	0.5948	7	0.4426	4	0.623	4	0.2758	3
Un-use of update software	0.7368	4	0.6617	3	0.4875	3	0.5532	16	0.2697	4
Designer lake experience	0.6684	11	0.5933	8	0.3966	9	0.5903	10	0.2341	9
Unskilled drawings	0.6766	8	0.6015	6	0.4070	7	0.545	18	0.2218	10
Mistakes in the contract	0.6632	12	0.5881	9	0.3900	10	0.6156	5	0.2401	6
Lack experience consultant	0.6714	9	0.5963	10	0.4003	8	0.5435	19	0.2176	11
Poor project management	0.7591	2	0.684	2	0.5192	2	0.5985	6	0.3108	2
Delay in the main changes	0.6446	14	0.5695	12	0.3671	12	0.5323	21	0.1954	16
Complication of the design	0.6387	16	0.5636	14	0.3600	15	0.5413	20	0.1948	17
Unsatisfactory data for design	0.6862	5	0.5071	22	0.3479	18	0.5933	8	0.2064	13
unskilled design team	0.6104	21	0.5353	20	0.3268	21	0.5316	22	0.1737	24
Misunderstanding of requirements	0.5807	23	0.5056	23	0.2936	23	0.5918	9	0.1738	23
Incomplete project design	0.6290	18	0.5539	16	0.3484	17	0.597	7	0.208	12
Unclear details in drawings	0.6312	17	0.5561	15	0.3510	16	0.577	12	0.2025	14
Project design get changed	0.6461	13	0.5799	11	0.3747	11	0.5026	24	0.1883	20
The consultant's staff not on site	0.6848	6	0.5354	19	0.3666	13	0.5041	23	0.1848	21
Delays in making design documents	0.6216	19	0.5465	18	0.3397	19	0.5851	11	0.1988	15

Note: SI = severity index, R = Rank, FI = frequency index, RV = risk value, DI = detectability index, RPN = risk priority number.

**Table 11:** Ranking of the contractor related delay causes.

Delay causes	SI	R	FI	R	RV	R	DI	R	RPN	R
Inadequate management	0.6989	6	0.6238	8	0.436	6	0.658	2	0.2869	3
Financial problems	0.7160	3	0.6409	5	0.4589	3	0.6468	4	0.2968	2
Delay in site mobilization	0.6810	11	0.548	22	0.3732	14	0.652	3	0.2433	9
Lack of experience	0.7108	4	0.6357	6	0.4518	4	0.5338	32	0.2412	10
Poor contractor's work	0.6290	21	0.5621	20	0.3535	22	0.6461	5	0.2284	14
Rework due to errors	0.6840	9	0.6186	10	0.4231	8	0.6409	6	0.2712	6
Poor financial control on site	0.6558	15	0.5978	12	0.3920	12	0.6357	7	0.2492	8
Ineffective project planning	0.6721	12	0.597	13	0.4013	9	0.6238	10	0.2503	7
Little production	0.6625	14	0.597	13	0.3955	11	0.597	17	0.2361	12
Sub-contractors delay	0.6967	7	0.6216	9	0.4330	7	0.6305	8	0.273	5
Insufficient methods	0.6089	29	0.5338	29	0.325	30	0.5323	34	0.173	32
Poor site organization	0.6483	16	0.5732	17	0.3716	15	0.5286	36	0.1965	24
Poor communication	0.6327	20	0.5576	21	0.3528	23	0.5212	37	0.1839	28
Unsuccessful planning	0.6394	19	0.5643	19	0.3608	20	0.5331	33	0.1923	26
Change of sub-contractors	0.6833	10	0.5212	33	0.3561	21	0.6216	11	0.2214	16
Difficulties in the financing	0.6149	25	0.5368	27	0.3301	29	0.6186	12	0.2042	18
Conflicts in schedule	0.5829	35	0.4773	39	0.2782	38	0.5978	16	0.1663	36
Unavailability of resources	0.5621	38	0.6468	3	0.3636	19	0.6141	13	0.2233	15
Low equipment-operator's	0.6141	26	0.539	25	0.331	28	0.6	15	0.1986	22
Poor quality	0.6037	31	0.5286	32	0.3191	33	0.6059	14	0.1934	25
Low productivity of labors	0.6476	17	0.5725	18	0.3707	16	0.5955	18	0.2208	17
Weak motivation	0.5844	34	0.5093	36	0.2976	36	0.5732	20	0.1706	34
Inaccessibility of utilities	0.5517	40	0.6186	10	0.3413	24	0.5874	19	0.2005	20
Effect of community	0.7212	2	0.6461	4	0.466	2	0.5167	38	0.2408	11
Accident during construction	0.6706	13	0.5955	15	0.3994	10	0.5725	21	0.2286	13
Delay in providing services	0.6112	28	0.5138	35	0.314	34	0.5621	23	0.1765	31
Lake of the experience	0.5673	36	0.4803	38	0.2725	39	0.5138	39	0.14	40
Unsuitable techniques	0.6186	24	0.5375	26	0.3325	27	0.5353	31	0.178	30
Unskilled project team	0.7056	5	0.6305	7	0.4449	5	0.6275	9	0.2792	4
Poor site management	0.7331	1	0.6580	1	0.4824	1	0.6669	1	0.3217	1
Insufficient site investigation	0.6238	22	0.5353	28	0.3339	26	0.5093	40	0.1701	35
Unsuitable guidelines	0.6059	30	0.5309	31	0.3216	32	0.5576	24	0.1794	29
Lack of materials	0.6119	27	0.5309	30	0.3248	31	0.5309	35	0.1724	33
Delay in the delivery	0.6855	8	0.5398	24	0.37	17	0.5368	30	0.1986	21
Damage of sorted material	0.5561	39	0.4669	40	0.2597	40	0.5442	26	0.1413	39
Shortage of labors	0.5881	33	0.5056	37	0.2973	37	0.548	25	0.1629	38
Inadequate material storage	0.5918	32	0.5167	34	0.3058	35	0.5398	27	0.1651	37
Low productivity	0.5636	37	0.652	2	0.3675	18	0.5375	29	0.1975	23
Changeable suppliers	0.6424	18	0.5874	16	0.3773	13	0.539	28	0.2034	19
Employment injuries	0.6193	23	0.5442	23	0.337	25	0.5643	22	0.1902	27

Note: SI = severity index, R = Rank, FI = frequency index, RV = risk value, DI = detectability index, RPN = risk priority number.

**Table 12:** Ranking of the security related delay causes.

Delay causes	SI	R	FI	R	RV	R	DI	R	RPN	R
War at the construction project site	0.727	2	0.727	2	0.529	2	0.617	15	0.327	7
IED blast on the way to the project	0.611	14	0.612	13	0.374	13	0.658	10	0.246	14
Local government official's corruption	0.705	7	0.704	7	0.497	7	0.716	8	0.356	5
Local tribal leader's interruption	0.723	3	0.726	3	0.525	3	0.692	9	0.364	3
Utilization of locally sourced materials	0.626	12	0.626	12	0.391	12	0.749	1	0.293	10
Mobilization works difficulties	0.717	5	0.717	5	0.514	5	0.739	3	0.380	2
Security officials from government	0.603	15	0.603	15	0.364	15	0.741	2	0.270	13
Local illegal armed people	0.736	1	0.736	1	0.541	1	0.641	11	0.347	6
Kidnap of contractor or engineering staff	0.679	9	0.679	9	0.461	9	0.624	14	0.288	12
Illegal taxes by armed groups	0.713	6	0.713	6	0.508	6	0.639	12	0.325	8
Provision of construction equipment	0.723	4	0.723	4	0.523	4	0.730	5	0.382	1
Transport of project teams to site	0.644	10	0.648	10	0.417	10	0.719	7	0.300	9
Supply of construction materials	0.702	8	0.702	8	0.493	8	0.726	6	0.358	4
Construction samples testing delays	0.628	11	0.628	11	0.394	11	0.736	4	0.290	11
Crimes of thieves along the way	0.614	13	0.609	14	0.374	14	0.627	13	0.234	15

Note: SI = severity index, R = Rank, FI = frequency index, RV = risk value, DI = detectability index, RPN = risk priority number.

Table 9 utilizes the Risk Priority Number (RPN) method to rank government-related causes of delay in Afghan construction projects. Each cause receives scores for its perceived severity, frequency of occurrence, and detectability. These scores are then combined into a single Risk Value (RV) to prioritize the most critical causes. The table highlights delay causes like late payments, difficulty obtaining site access, corruption, bureaucracy, and awarding projects to the lowest bidder as the most problematic based on their high RV scores. While RV offers a comprehensive picture, the table also provides individual rankings for severity, frequency, and detectability of each cause, allowing for a more nuanced understanding of their contribution to delays.

Table 10 ranks consultant-related causes of delay in Afghan construction projects. Similar to government causes, high Risk Value (RV) scores indicate the most problematic issues. These include delays in document reviews, poor data collection during surveys, outdated software use, inadequate project management, unskilled design teams, and incomplete project designs. The table delves deeper by providing individual rankings for severity, frequency, and detectability of each consultant-related cause, offering a more comprehensive understanding of their impact on project timelines.

This table highlights 40 contractor-related delay causes for Afghan projects. Maximum offenders include inadequate regulation (most impactful but easily detected), financial woes, and late site mobilization. Inexperienced staff, construction errors, and poor planning pose high risks. While delays with subcontractors and low productivity are common, their impact is mitigated by detectability. Interestingly, community issues and accidents have high severity but are less frequent.

Table 12. Determines Security related delay causes in Afghan construction, with local militia threats, war, and tribal interference topping the list. These highly impactful issues, though less frequent, demand immediate attention. Corruption to officials, kidnapping, and illegal taxes pose significant risks as well. While delays in material and equipment supply are common, their impact is mitigated by detectability. Interestingly, local government security involvement has low severity despite being frequent, suggesting inefficiencies rather than security breaches.

### 4.2 Analysis on the root causes of delay

**Fig. 2:**
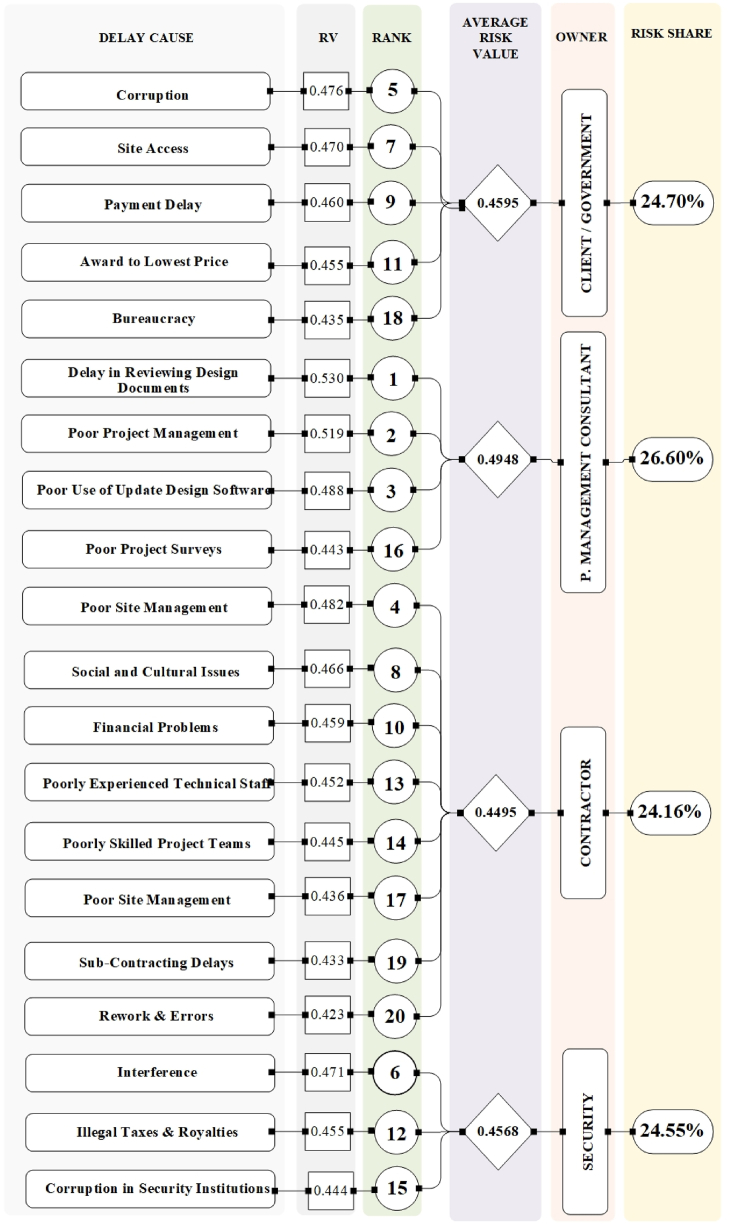
Analysis of top 20 delay causes based on risk value (RV).

**Fig. 3:**
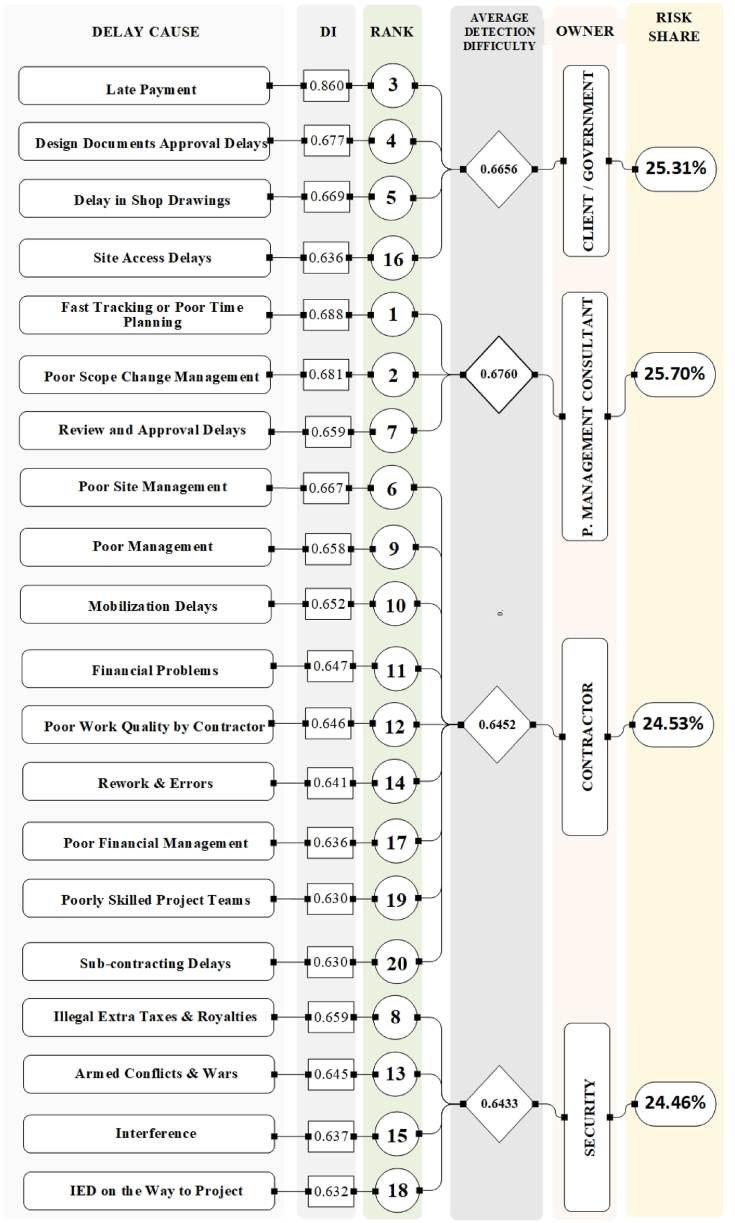
Analysis of top 20 delay causes based on detecting probability (DI).

**Fig. 4:**
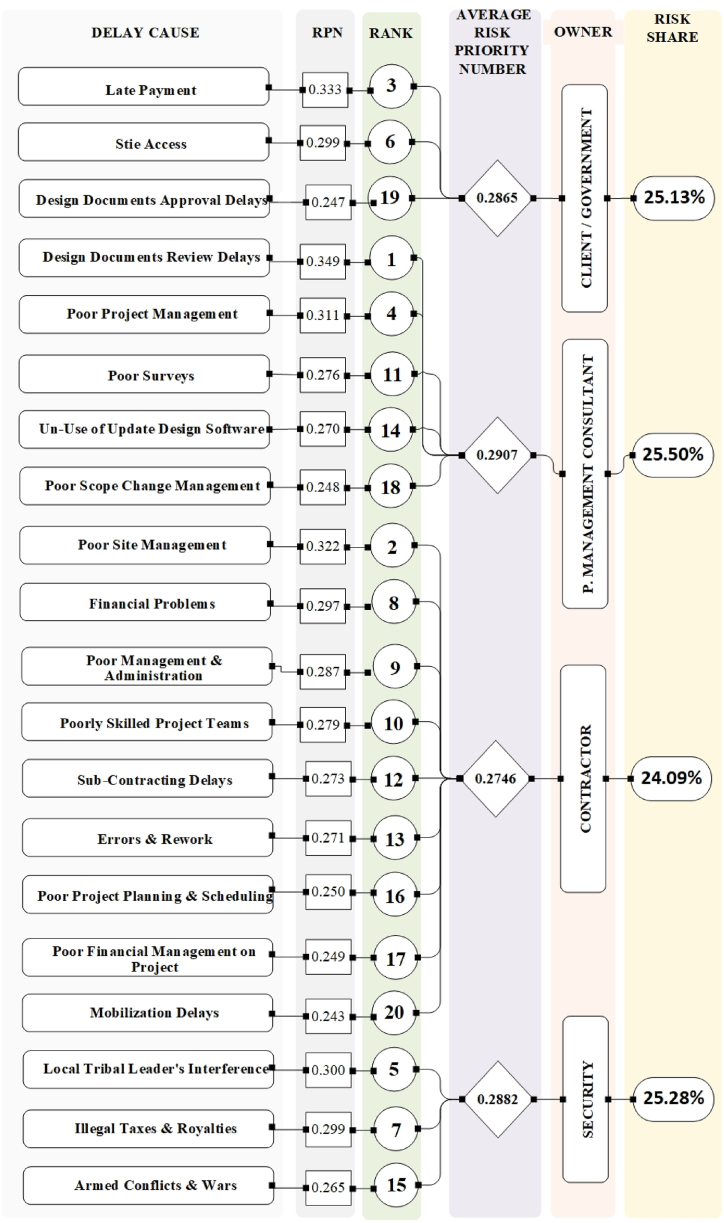
Analysis of top 20 delay causes based on risk priority number (RPN).

**Fig. 5:**
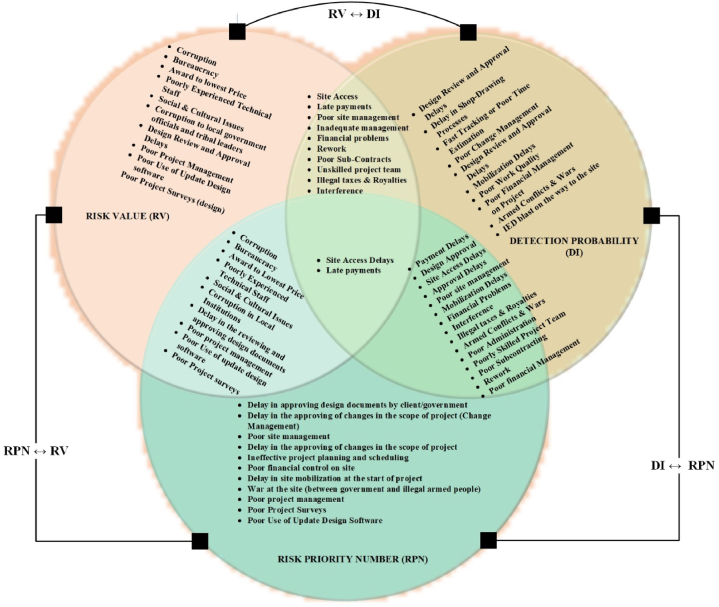
Combination of top 20 delay causes.

This section analyzes the root causes contributing to delays in Afghan construction projects, based on three key indicators: Risk Value (RV), Detectability Index (DI), and Risk Priority Number (RPN). Fig. 2, Fig. 3, Fig. 4, Fig. 5 visually represent the ranking of each delay cause's impact, along with the average values for each main category (government, consultant, contractor, and security). These rankings are presented following the Zhang et al. (2020) method [68].

The selection of the top 20 delay causes in Fig. 2, Fig. 3, Fig. 4, Fig. 5 was based on the ranking methodology detailed in Section 4.1. The ranking process utilized the Risk Priority Number (RPN) method, which considers severity, frequency, risk value (RV), and detectability (DI). From the total of 97 delay causes identified in Table 7, Table 8, Table 9, Table 10, the top 20 causes were selected based on their highest RPN, RV, and DI values. This approach ensured that the most critical delay causes were highlighted in the analysis.

Fig. 5 combines the findings from Fig. 2, Fig. 3, Fig. 4, providing a comprehensive overview of the top 20 delay causes. By visually representing which causes appear in the top 20 rankings for each of the three indices (RV, DI, and RPN), the figure reveals valuable insights. It identifies high-risk, hard-to-detect causes like "corruption" and "awarding low bids," as well as frequent and severe causes such as "poor planning" and "financial control issues" that require immediate attention. Importantly, the figure highlights "providing site access to the contractor" and "late payments from the government to the contractor" as the only two causes present in all three indices, emphasizing their critical role in causing delays and necessitating targeted mitigation strategies. This visual representation provides a valuable tool for understanding the complex interplay of factors contributing to construction project delays in Afghanistan.

A comprehensive analysis using the Risk Priority Number (RPN) reveals significant contributions from all four categories (consultants, government, contractors, security) to project delays. Fig. 2, Fig. 3, Fig. 4, Fig. 5 offer a detailed breakdown of these contributions. While all categories exhibit an average Risk Value (ARV) around 0.45, indicating a substantial impact on project timelines, consultants stand out with the highest average ARV. However, a silver lining exists – their delays might be easier to mitigate due to a higher Detectability Index (DI). This suggests that while consultant-related delays are frequent and severe, they are also more readily identifiable, allowing for quicker intervention.

Government delays, on the other hand, are generally the easiest to identify due to their high Detectability Index. However, their overall impact might be comparable to consultant delays based on the Risk Value. When prioritizing mitigation efforts, the RPN provides a more nuanced view. RPN considers both the severity/frequency of a delay cause and its detectability. While the overall risk shares across categories might be similar, the RPN ranking can differ slightly from the ARV/DI rankings. For instance, consultant delays, though exhibiting high severity and frequency, might be addressed faster due to their higher DI compared to other categories.

### 4.3 Comparing key delay causes in Afghanistan and other conflict-affected countries

The comparative analysis of construction project delays in Afghanistan, Ukraine, Pakistan, Palestine, and Iraq highlights both shared and distinct delay causes. Common challenges include political instability, corruption, security threats, and economic instability. Afghanistan, however, faces additional hurdles due to tribal interference, exacerbating corruption and bureaucratic inefficiencies. Security threats and ongoing conflicts delay construction across all countries, though Afghanistan experiences more localized disruptions from warlords, while Palestine faces broader blockades. Economic instability, such as inflation and limited financing, is a shared issue, with Afghanistan's delays compounded by late payments from government, a problem also seen in Ukraine and Palestine. Resource shortages are prevalent, particularly in Afghanistan and Palestine due to conflict and trade restrictions.

**Fig. 6:**
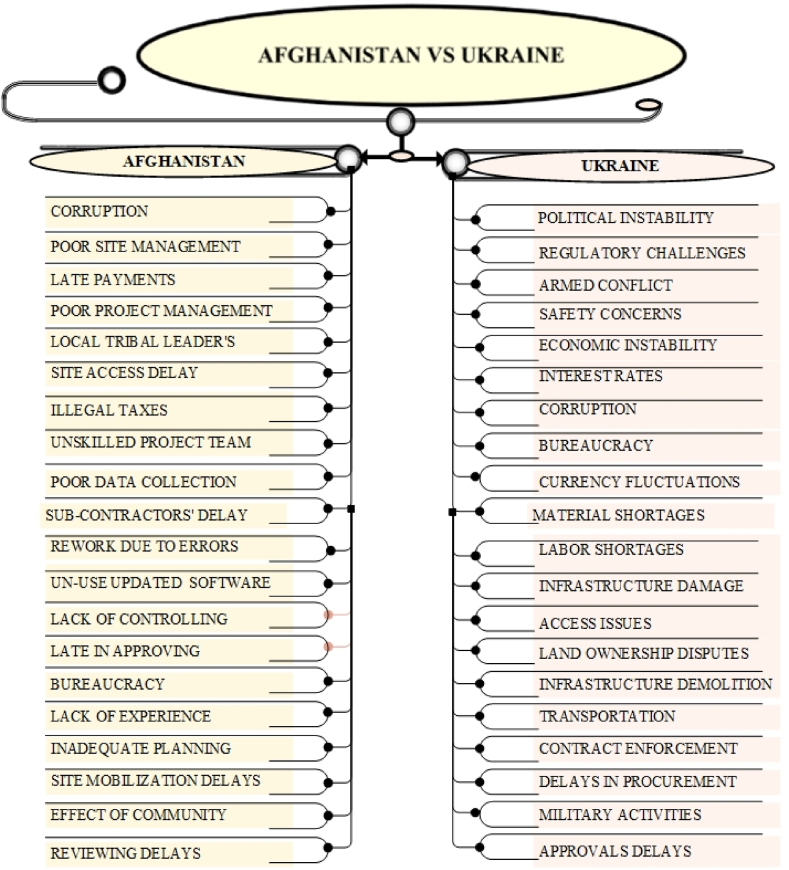
key delay causes in construction projects in Afghanistan and Ukraine.

**Fig. 7:**
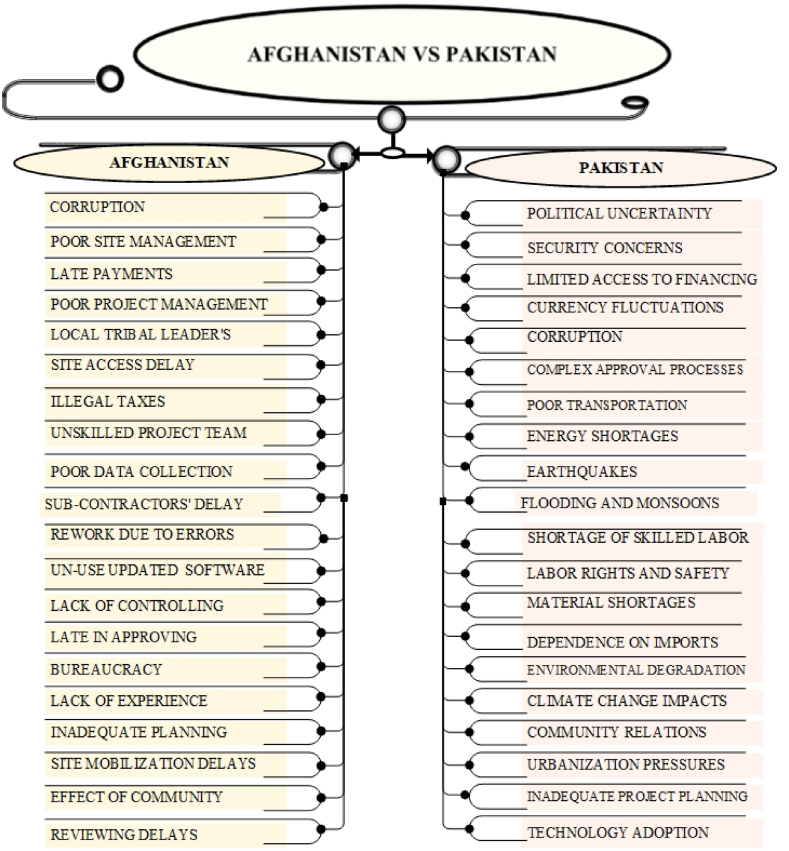
Key delay causes in construction projects in Afghanistan and Pakistan.

**Fig. 8:**
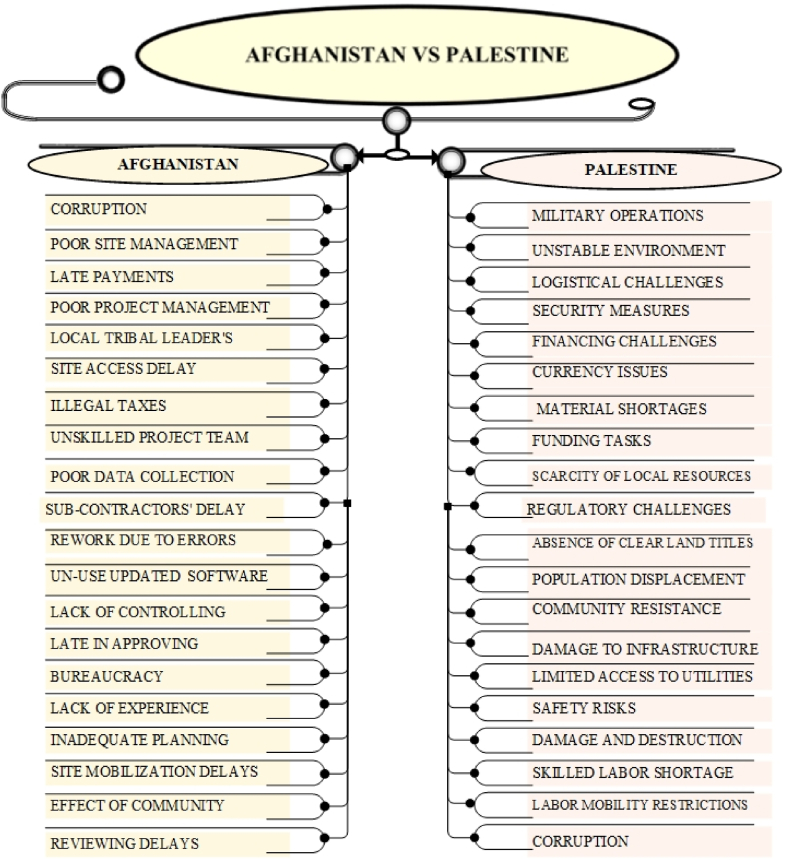
Key delay causes in construction projects in Afghanistan and Palestine.

**Fig. 9:**
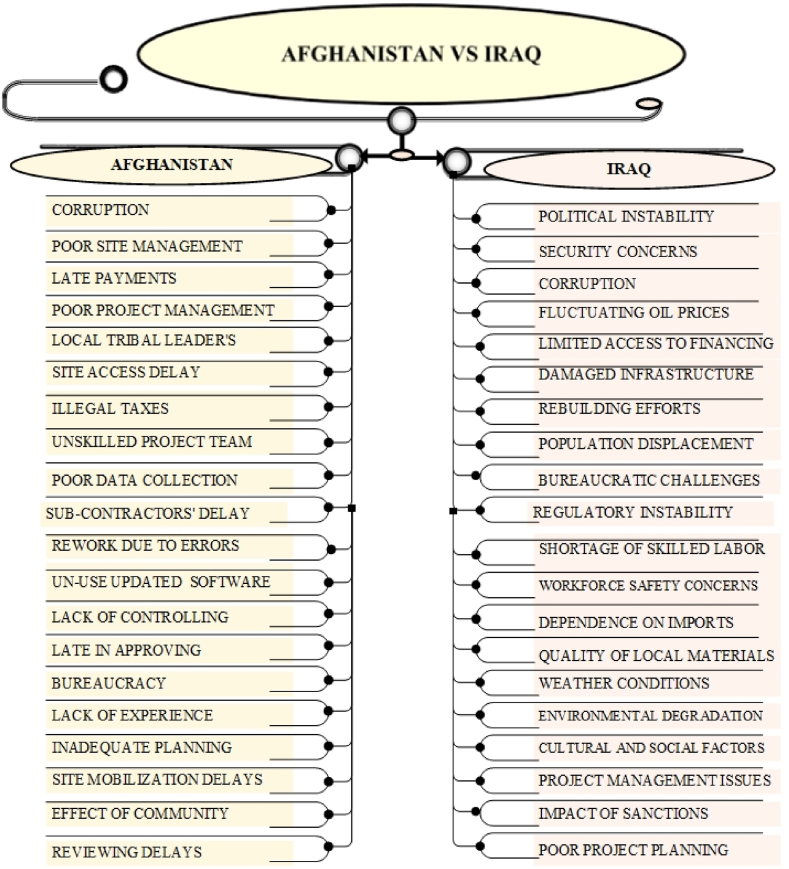
key delay causes in construction projects in Afghanistan and Iraq.

Distinct causes include Afghanistan's conflict-driven delays and tribal dynamics, while Ukraine deals more with government-driven delays, and Pakistan grapples with political instability and economic fluctuations. Palestine's construction sector suffers from restricted access and blockades, while Iraq, with a stable oil-based economy, faces less severe delays. The findings emphasize the need for tailored solutions, especially in Afghanistan's unique context of tribal dynamics, corruption, and underdevelopment, requiring targeted interventions to mitigate delays and improve construction outcomes. The statistics and detailed descriptions of the comparative analysis are shown in Fig. 6, Fig. 7, Fig. 8, Fig. 9.

Both Afghanistan and Ukraine encounter a complex interplay of factors that hinder the successful execution of construction projects. Shared challenges include political instability, creating an unpredictable environment for project planning and execution. Corruption is pervasive in both countries, leading to delays in approvals, increased costs, and reduced transparency. Additionally, both nations grapple with infrastructure deficiencies, such as inadequate transportation and energy networks, which impede project progress and increase costs. Economic instability, characterized by inflation, currency fluctuations, and limited access to financing, is another common challenge affecting project viability in both countries. While sharing common challenges, Afghanistan and Ukraine exhibit distinct characteristics in the factors affecting construction projects. Afghanistan's construction sector is profoundly impacted by ongoing conflict, with security threats, including terrorism and local insurgencies, posing significant risks. In contrast, while Ukraine faces security challenges, particularly in eastern regions, the overall security situation is relatively less volatile. Afghanistan's reliance on foreign aid and a less developed economy exacerbate its construction challenges, while Ukraine's economy, though facing difficulties, is more diversified. Furthermore, the role of tribal authorities and customary laws in Afghanistan introduces unique complexities compared to Ukraine's more established legal and governance framework.

Afghanistan and Pakistan appearance substantial construction delays due to financial instability, infrastructure deficiencies, and labor market issues. Each country struggles with resource shortages that impact project momentum, and both deal with inadequate infrastructure that disrupts construction operations. Labor shortages and issues with skilled workforce affect project quality and efficiency in both nations. Additionally, environmental and cultural factors, including natural disasters and social disputes, contribute to delays in construction projects. In Afghanistan, delays are exacerbated by ongoing conflict, security issues, and interference from local tribal leaders, leading to bureaucratic inefficiencies and disrupted site management. Economic challenges stem from late payments and overall instability. Pakistan, however, experiences delays due to political instability, frequent government changes, and economic issues like inflation and currency fluctuations. Infrastructure problems differ as well, with Pakistan facing poor transportation networks and energy shortages, while Afghanistan contends with inadequate site management. Environmental and cultural challenges are also distinct, with Afghanistan dealing with resource availability and site management, and Pakistan facing natural disasters and urbanization pressures.

Construction projects in both Afghanistan and Palestine encounter substantial delays due to political instability and conflict. In each region, security concerns arising from ongoing conflict or military operations disrupt project activities and elevate risks. Economic constraints further complicate the situation in both countries, with financial instability, difficulties in securing funds, and limitations on accessing international resources. Resource shortages, driven by conflict in Afghanistan and blockades in Palestine, also hinder construction progress, while bureaucratic inefficiencies and complex regulatory environments exacerbate delays in both regions. In Afghanistan, the primary challenges include interference from local tribal leaders and extensive bureaucratic inefficiencies, which are compounded by ongoing armed conflict and security issues. Financial difficulties such as late payments from clients and economic instability further impact project timelines. In contrast, Palestine faces a unique set of challenges related to the Israeli-Palestinian conflict, including restricted access due to checkpoints and border closures, which severely affect construction efforts. The blockade and trade restrictions in Palestine limit the import of essential construction materials, adding to project delays and increasing costs. Additionally, while both regions experience legal and regulatory obstacles, Palestine deals with complex land ownership issues and a protracted permit process, especially in areas controlled by Israel, which can lead to significant project modifications or cancellations.

Both Afghanistan and Iraq face significant challenges in their construction sectors due to political instability, security threats, and economic difficulties. The ongoing conflicts in each country create an environment where construction projects are frequently disrupted by security issues, including attacks and instability. Economic challenges, such as fluctuations in funding and financial constraints, further complicate project execution in both nations. Additionally, both countries struggle with corruption and bureaucratic inefficiencies, which hinder timely project approvals and implementation. Afghanistan's construction sector is profoundly affected by tribal dynamics and pervasive conflict, leading to frequent delays caused by local interference and war-related disruptions. Its relatively underdeveloped economy exacerbates these issues, creating a more challenging environment for project execution. In contrast, Iraq, despite its significant conflict-related issues, benefits from a more stable oil-based economy, which provides some economic resilience against global price fluctuations. Iraq's infrastructure, although damaged, is more developed compared to Afghanistan's, potentially easing issues related to basic infrastructure like power supply and transportation. Additionally, while both countries face corruption and administrative inefficiencies, Afghanistan's governance is further complicated by the influence of tribal and religious authorities, whereas Iraq operates under a more centralized governance structure.

## 5 Result and discussion

### 5.1 Result

The analysis of delay causes in Afghan construction projects reveals a complex interplay of the root causes contributing to project delays. To prioritize these causes, the Risk Priority Number (RPN) method was employed, ranking causes based on their severity, frequency, and detectability. A comparative analysis of delay causes in Afghanistan and other conflict-affected countries—Ukraine, Pakistan, Palestine, and Iraq—highlights both shared and distinct challenges. Common challenges include political instability, corruption, security threats, and economic instability. However, Afghanistan faces unique challenges due to tribal interference, localized conflicts, and severe resource shortages.

Security threats, corruption, and logistical hurdles emerge as significant contributors to project delays. Security threats, such as terrorism and local militia interference, disrupt project activities and increase costs. Corruption at various levels hinders project approvals and execution. Logistical hurdles, including infrastructure deficiencies, limited access to resources, and supply chain disruptions, further contribute to delays. To address these challenges, tailored strategies are essential. Strengthening security measures, enhancing project management practices, addressing corruption, improving infrastructure, building local capacity, and facilitating access to finance are key areas for intervention.

Implementing effective security measures to protect construction sites and personnel is crucial. Enhancing project management practices involves improving planning, scheduling, and coordination to minimize delays. Addressing corruption requires implementing anti-corruption measures and promoting transparency in government processes. Investing in infrastructure development is essential to support construction projects and improve access to resources. Building local capacity by enhancing the skills and capabilities of local contractors and project managers is another important strategy. Finally, facilitating access to affordable financing options for construction projects can help mitigate financial constraints. By implementing these strategies, Afghanistan can address the root causes of construction project delays and improve project outcomes. This will contribute to the nation's development and economic growth.

This research provides valuable insights for policymakers, practitioners, and researchers seeking to bolster the performance of Afghanistan's construction sector. By understanding the root causes influencing project delays and implementing appropriate mitigation strategies, Afghanistan can enhance project outcomes and promote sustainable development.

### 5.2 Discussion

The research revealed a multifaceted landscape where various interrelated causes contribute to significant project setbacks. The findings highlight that while common challenges, such as political instability, corruption, and security threats, are pervasive across conflict-affected countries, Afghanistan stands out due to its unique socio-political dynamics and environmental context. The utilization of the Risk Priority Number (RPN) method has provided a structured framework for assessing and prioritizing the causes of construction project delays. The data indicates that security threats, particularly those stemming from local militia activity and ongoing conflict, rank among the most impactful cause. These threats not only lead to direct disruptions but also impose indirect costs that exacerbate delays due to increased security measures and affected workforce morale. Corruption remains a pervasive issue influencing project timelines, with bureaucratic inefficiencies and unethical practices often hindering timely project execution.

A comparative analysis with similar conflict-affected countries reveals that while many of the identified delay causes overlap, Afghanistan's situation is uniquely compounded by elements such as local tribal dynamics and a less developed economic infrastructure. In contrast, countries like Iraq benefit from a more stable economy driven by oil, reducing the severity of certain delays. This emphasizes the necessity for tailored interventions that address Afghanistan's specific challenges rather than solely relying on broader regional solutions. The interplay of these causes suggests that a one-size-fits-all approach to solving construction delays is ineffective; instead, strategies must be context-specific and sensitive to local conditions.

The findings underscore the necessity of implementing a multi-faceted approach to address the root causes of delays. Key implications for policymakers and practitioners include enhancing security measures by collaborating with local security forces and community leaders to create a safer working environment. Addressing corruption requires establishing transparent processes for project approvals and oversight to mitigate the negative effects of unethical practices. Furthermore, training and capacity-building initiatives for local project managers and contractors can significantly improve operational efficiency, thus minimizing delays.

Investing in better infrastructure is vital for improving logistics and supply chains, which address one of the core facilitators of delays. This could also involve public-private partnerships that leverage resources effectively for both construction and infrastructure improvement. Another critical area is financial accessibility; providing affordable financing options for construction projects can alleviate some of the economic constraints contributing to delays. This may include targeted loans or grants for contractors that demonstrate a capacity to execute projects efficiently, leading to enhanced overall project delivery.

By implementing effective security measures, Afghanistan can create a safer working environment for construction projects. Enhancing project management practices involves improving planning, scheduling, and coordination to minimize delays. Addressing corruption requires implementing anti-corruption measures and promoting transparency in government processes. Investing in infrastructure development is crucial for improving logistics and supply chains. Building local capacity by enhancing the skills and capabilities of local contractors and project managers is another important strategy. Finally, facilitating access to affordable financing options for construction projects can help alleviate financial constraints.

This research provides valuable insights for policymakers, practitioners, and researchers seeking to bolster the performance of Afghanistan's construction sector. By understanding the root causes of delays and implementing appropriate mitigation strategies, Afghanistan can enhance project outcomes and promote sustainable development.

The findings underscore the importance of tailored approaches to address the specific challenges faced by Afghanistan. While lessons from other countries can provide valuable insights, a one-size-fits-all approach is ineffective. By implementing targeted interventions, Afghanistan can mitigate delays, improve project effectiveness, and contribute to its development.

## 6 Conclusion and recommendations

### 6.1 Conclusion

This research offers a comprehensive examination of the root causes contributing to construction project delays in Afghanistan, employing a mixed-methods approach that integrates quantitative analysis via the Risk Priority Number (RPN) method and qualitative insights from key stakeholders. The findings highlight the complex interplay of delay causes across various categories—Government, Consultants, Contractors, and Security—underscoring the need for a holistic approach to mitigation. Key causes such as security threats, corruption, logistical hurdles, and inefficiencies in project management emerged as significant contributors to delays. The study's novel focus on security-related delays in Afghanistan, a previously underexplored area, provides valuable insights into how these issues uniquely impact project timelines. The comparative analysis with other conflict-affected countries also sheds light on shared and distinct challenges, laying the foundation for identifying global best practices.

Proactive strategies, such as strengthening communication protocols, implementing robust security measures, addressing financial and managerial deficiencies among contractors, and improving project management practices, are essential to mitigating these delays. Tailored interventions—such as enhanced infrastructure, anti-corruption measures, and capacity-building initiatives—are critical for addressing Afghanistan's unique socio-political and logistical challenges.

This research not only advances understanding of the root causes of construction project delays but also provides actionable recommendations for stakeholders, including policymakers, project managers, and contractors. By implementing these strategies, Afghanistan can improve its construction project outcomes, contributing to broader development goals and economic growth. The findings also pave the way for future studies to quantify the economic impact of these delays, explore the cost-effectiveness of mitigation strategies, and conduct further comparative analyses with other conflict-affected regions to enhance global understanding of how to tackle similar challenges.

This study provides valuable insights into the root causes influencing construction project delays in Afghanistan. However, it is important to acknowledge certain limitations. The study relied on data collected through questionnaires and interviews, which may have inherent limitations in terms of sample size, response rates, and the potential for bias. Additionally, the findings may not be fully generalizable to all construction projects in Afghanistan or other conflict-affected countries due to variations in project size, complexity, and specific circumstances. Furthermore, the dynamic security and political landscape in Afghanistan and other conflict-affected countries means that the findings may not be applicable to future projects or conditions. Finally, while the study analyzed a range of causes, it is possible that other factors not included in the analysis may also play a significant role. Despite these limitations, the study provides valuable insights that can inform policy decisions, project management practices, and future research efforts to address the challenges of construction delays in complex environments.

### 6.2 Recommendations

Based on the findings of this research on the root causes of construction project delays in Afghanistan, the following recommendations are proposed to mitigate delays and enhance project outcomes.

1. Strengthening Security and Infrastructure: Security threats and logistical hurdles are major causes of delays in Afghan construction projects. It is recommended that construction stakeholders collaborate with local security forces and community leaders to protect project sites and ensure the safety of personnel. Additionally, investing in infrastructure development, such as roads and communication networks, will improve resource accessibility and streamline project execution.
2. Improving Project Management and Capacity Building: Effective project planning, scheduling, and resource management are essential to reducing delays. This can be achieved by adopting advanced project management tools and methodologies. Additionally, enhancing the skills and capacity of local contractors and project managers through training programs will promote better project execution and minimize delays caused by mismanagement or insufficient expertise.
3. Addressing Corruption and Streamlining Bureaucracy: Corruption and bureaucratic inefficiencies significantly contribute to project delays. To combat this, government agencies and private stakeholders should implement anti-corruption measures and transparent processes for project approvals and oversight. Streamlining approval processes through digital workflows and setting clear timelines for payments and design approvals can reduce administrative delays.
4. Facilitating Access to Finance: Financial constraints often result in project delays. It is essential to improve access to affordable financing options for contractors and developers. Targeted loans or grants from government institutions or financial bodies can provide the necessary capital for projects to proceed without delay, particularly for contractors with proven efficiency.
5. Fostering Stakeholder Collaboration: Enhancing communication and collaboration between key stakeholders—clients, contractors, consultants, and government entities—is vital to addressing delays early on. Regular meetings, transparent decision-making processes, and clear communication channels will ensure that potential delays are identified and mitigated before they escalate, leading to more efficient project execution.

By focusing on these key areas, Afghanistan's construction sector can significantly improve project delivery and contribute to the nation's economic and infrastructure development.

## CRediT authorship contribution statement

**Mohammad Basheer Ahmadzai:** Writing – review & editing, Writing – original draft, Validation, Software, Methodology, Investigation, Formal analysis, Data curation, Conceptualization. **Kunhui Ye:** Supervision.

## Data availability statement

The first author can provide the data from this study upon request.

## Declaration of competing interest

The authors declare that they have no known competing financial interests or personal relationships that could have appeared to influence the work reported in this paper.

## References

[1] Dara H.M., Raut A., Adamu M., Ibrahim Y.E., Ingle P.V. (2024). Reducing non-value added (NVA) activities through lean tools for the precast industry. Heliyon.

[2] Yang Y., Wang Y., Easa S.M., Yan X. (2023). Risk factors influencing tunnel construction safety: structural equation model approach. Heliyon.

[3] Awasho T.T., Alemu S.K. (2023). Assessment of public building defects and maintenance practices: cases in Mettu town, Ethiopia. Heliyon.

[4] Koohathongsumrit N., Meethom W. (2024). Risk analysis in underground tunnel construction with tunnel boring machines using the Best–Worst method and data envelopment analysis. Heliyon.

[5] Maboudi Reveshti A., Khosravirad E., Rouzbahani A.K., Fariman S.K., Najafi H., Peivandizadeh A. (2024). Energy consumption prediction in an office building by examining occupancy rates and weather parameters using the moving average method and artificial neural network. Heliyon.

[6] Musarat M.A., Alaloul W.S., Liew M.S. (2024). Incorporating inflation rate in construction projects cost: forecasting model. Heliyon.

[7] Rivera L., Baguec H., Yeom C. (2020).

[8] Nawaz A., Chen J., Su X., Zahid Hassan H.M. (2022). Material based penalty-cost quantification model for construction projects influencing waste management. Front. Environ. Sci..

[9] Alshibani A., Julaih M., Adress A., Alshamrani O., Almaziad F. (2023). Identifying and ranking the root causes of schedule delays in oil and Gas pipeline construction projects. Energies.

[10] Japee G.P. (October, 2022).

[11] Bukhari S.R.A., Nasir A.R., Greco R., Mollo L. (2024). The impact of COVID-19 on construction project performance: a case study in Pakistan. Covid.

[12] Nyaga Githae B., Hagir H., Alowo R. (2024). An investigation on construction project development planning delays in South Africa. Buildings.

[13] Bageis A.S. (2024). An investigation into the causes of payment delays and deliberate delay tactics in public construction projects in Saudi arabia. Buildings.

[14] Karimi S., Piroozfar P. (May, 2022).

[15] Zadran B.G., Elham F.A., Rahim A., Hamid A. (2023).

[16] Alnaser A.A., Alsanabani N.M., Al-Gahtani K.S. (2023). BIM impact on construction project time using system dynamics in Saudi arabia's construction. Buildings.

[17] Eskander R.F.A. (2018). Risk assessment influencing factors for Arabian construction projects using analytic hierarchy process. Alexandria Eng. J..

[18] Nguyen D.T., Le-Hoai L., Basenda Tarigan P., Tran D.H. (2022). Tradeoff time cost quality in repetitive construction project using fuzzy logic approach and symbiotic organism search algorithm. Alexandria Eng. J..

[19] Wafa W., Sharaai A.H., Sahak K., Afghan F.R. (September, 2023). A CASE STUDY.

[20] Science E. (2023).

[21] Kim K.J., Han B., Park M.S., Kim K., Kim E.W. (2022). Application issues of impacted as-planned schedule for delay analysis. Buildings.

[22] Alenazi E., Adamu Z., Al-otaibi A. (2022). Contemporary Saudi construction projects. Buildings.

[23] Çevikbaş M., Işık Z. (2021). An overarching review on delay analyses in construction projects. Buildings.

[24] Koulinas G.K., Xanthopoulos A.S., Tsilipiras T.T., Koulouriotis D.E. (2020). Schedule delay risk analysis in construction projects with a simulation-based expert system. Buildings.

[25] Sepasgozar S.M.E., Karimi R., Shirowzhan S., Mojtahedi M., Ebrahimzadeh S., McCarthy D. (2019). Delay causes and emerging digital tools: a novel model of delay analysis, including integrated project delivery and PMBOK.

[26] El-khalek H.A., Aziz R.F., Morgan E.S. (2019). Identification of construction subcontractor prequalification evaluation criteria and their impact on project success. Alexandria Eng. J..

[27] Abdel-Khalek H.A., Aziz R.F., Abdellatif I.A. (2019). Prepare and analysis for claims in construction projects using Primavera Contract Management (PCM). Alexandria Eng. J..

[28] Alsuliman J.A. (2019). Causes of delay in Saudi public construction projects. Alexandria Eng. J..

[29] Duary A. (2022). Advance and delay in payments with the price-discount inventory model for deteriorating items under capacity constraint and partially backlogged shortages. Alexandria Eng. J..

[30] Abdel Khalek H.A., Aziz R.F., Abdeen A.H. (2018). Identify and prioritize the major influencing causes of automated concrete mixing system for mega construction projects using analytic hierarchy process. Alexandria Eng. J..

[31] Abdelmalek D. (2024). Groundwater quality assessment using revised classical diagrams and compositional data analysis (CoDa): case study of Wadi Ranyah, Saudi Arabia. J. King Saud Univ. Sci..

[32] Niazi G.A., Painting N. (2017). Significant factors causing cost overruns in the construction industry in Afghanistan. Procedia Eng..

[33] Ahmadzai M.B., Ye K. (2024). Modelling the impacts of security on construction delays: a case of Afghanistan. Heliyon.

[34] Shoaib A., Ariaratnam S. (2016). A study of socioeconomic impacts of renewable energy projects in Afghanistan. Procedia Eng..

[35] Koohathongsumrit N., Meethom W. (2024). Risk analysis in underground tunnel construction with tunnel boring machines using the Best–Worst method and data envelopment analysis. Heliyon.

[36] Alsugair A.M., Al-Gahtani K.S., Alsanabani N.M., Hommadi G.M., Alawshan M.I. (2024). An integrated DEMATEL and system dynamic model for project cost prediction. Heliyon.

[37] Galarraga I., Abadie L.M., Standfuss T., Ruiz-Gauna I., Goicoechea N. (2024). An approximation of flights, delays and costs for different forecast scenarios: a backcasting exercise. Heliyon.

[38] Datta S.D., Islam M., Rahman Sobuz M.H., Ahmed S., Kar M. (2024). Artificial intelligence and machine learning applications in the project lifecycle of the construction industry: a comprehensive review. Heliyon.

[39] Desse E.M., Mengesha W.J. (2024). Predicting construction cost under uncertainty using grey-fuzzy earned value analysis. Heliyon.

[40] Abkar M.M.A., Yunus R., Gamil Y., Albaom M.A. (2024). Enhancing construction site performance through technology and management practices as material waste mitigation in the Malaysian construction industry. Heliyon.

[41] Li L. (2024). The potential of construction robotics to reduce airborne virus transmission in the construction industry in the UK and China. Heliyon.

[42] Alqahtani F.K., Alsaud M., Al-Dossary S., Sherif M., Abotaleb I.S., Mohamed A.G. (2024). Evaluation of insurance policies in the Saudi Arabian construction contracts. Heliyon.

[43] León-Romero L.P., Aguilar-Fernández M., Luque-Sendra A., Zamora-Polo F., Francisco-Márquez M. (2024). Characterization of the information system integrated to the construction project management systems. Heliyon.

[44] Liu J., Chen Y., Wang W., Hao C., Cai F., Teng L. (2024). Heliyon Impact analysis of dust evolution pattern and determination of key ventilation parameters in highland highway construction tunnels. Heliyon.

[45] Akıner M.E., Akıner İ., Yitmen İ. (2024). Predicting the critical organizational behavior and culture of the Turkish construction industry's occupational groups for determining the success of the construction business. Heliyon.

[46] Karimi S., Zhakfar Z., Sarwary M.I. (2020). Study of excessive bureaucracy in construction projects causes of low level of competition and lengthy tendering process A case study of Afghanistan. Int. J. Eng. Adv. Technol..

[47] Khairullah N.H., Hilal M.A., Burhan A.M. (2023). J. Eng..

[48] Al L. (April, 2023).

[49] Vahedi Nikbakht M., Gheibi M., Montazeri H., Yeganeh Khaksar R., Moezzi R., Vadiee A. (2024). Identification and ranking of factors affecting the delay risk of high-rise construction projects using AHP and VIKOR methods. Infrastructures.

[50] Hussain S., Zhu F., Ali Z., Xu X. (2017). Rural residents' perception of construction project delays in Pakistan. Sustain. Times.

[51] Banobi E.T., Jung W. (2019). Causes and mitigation strategies of delay in power construction projects: gaps between owners and contractors in successful and unsuccessful projects. Sustain. Times.

[52] Schuldt S.J., Nicholson M.R., Adams Y.A., Delorit J.D. (2021). Weather-related construction delays in a changing climate: a systematic state-of-the-art review. Sustain. Times.

[53] Sabboubeh H.N., Farrell P., Osman Y. (2019). “Establishing sustainable construction in war zones ; Palestine as a case study establishing sustainable construction in war zones. Palestine as a Case Study.

[54] Dmaidi N., Mahamid I., Shweiki I. (2016). Identifying the critical problems of construction contracting management in Palestine.

[55] Chen Y.-T., Yang Y.-Y., Chen Y.-H. (2023). Assessing the COVID-19 impact of projects under construction with Monte Carlo simulation. Architecture.

[56] Sjögren Novikov Hans, Department of Business Administration (2023). The Invasion of Ukraine and its Effects on the Swedish Construction Sector.

[57] Tokarski S., Magdziarczyk M., Smoliński A. (2024). An analysis of risks and challenges to the polish power industry in the year 2024. Energies.

[58] Damaševičius R., Zailskaitė-Jakštė L. (2023). The impact of a national crisis on research collaborations: a scientometric analysis of Ukrainian authors 2019–2022. Publications.

[59] Shpak N., Kuzmin O., Melnyk O., Ruda M., Sroka W. (2020). Implementation of a circular economy in Ukraine: the context of European integration. Resources.

[60] Ayat M., Rehman H., Qureshi S.M. (2021). Assessing the causes of project overruns in tunnel construction projects in Pakistan. Int. J. Constr. Manag..

[61] Hasmori M.F., Memon M.H. (2023).

[62] Zafar I., Yousaf T., Ahmed D.S. (2016). Evaluation of risk factors causing cost overrun in road projects in terrorism affected areas Pakistan – a case study. KSCE J. Civ. Eng..

[63] Araújo-Rey C., Sebastián M.A. (2021). An approach to the analysis of causes of delays in industrial construction projects through planning and statistical computing. Sustain. Times.

[64] Khahro S.H., Shaikh H.H., Zainun N.Y., Sultan B., Khahro Q.H. (2023).

[65] Anugerah A.R., Muttaqin P.S., Trinarningsih W. (2022). Social network analysis in business and management research: a bibliometric analysis of the research trend and performance from 2001 to 2020. Heliyon.

[66] Tafesse S., Girma Y.E., Dessalegn E. (2022). Analysis of the socio-economic and environmental impacts of construction waste and management practices. Heliyon.

[67] Tessema A.T., Alene G.A., Wolelaw N.M. (2022). Assessment of risk factors on construction projects in gondar city, Ethiopia. Heliyon.

[68] Okon E.M. (2021). Systematic review of climate change impact research in Nigeria: implication for sustainable development. Heliyon.

[69] Noureen S., Mahmood Z. (2022). The effects of trade cost components and uncertainty of time delay on bilateral export growth. Heliyon.

[70] Maqbool R., Rashid Y., Altuwaim A., Tariq M., Oldfield L. (2023). Coping with skill shortage within the UK construction industry : scaling up training and development systems. Ain Shams Eng. J..

[71] Khodeir L.M., Nabawy M. (2019). Identifying key risks in infrastructure projects – case study of Cairo Festival City project in Egypt. Ain Shams Eng. J..

[72] Khodeir L.M., El A. (2019). Examining the role of value management in controlling cost overrun [ application on residential construction projects in Egypt ]. Ain Shams Eng. J..

[73] Dixit S., Mandal S.N., V Thanikal J., Saurabh K. (2019). Evolution of studies in construction productivity : a systematic literature review ( 2006 – 2017). Ain Shams Eng. J..

[74] Abdelkhalek H.A., Refaie H.S., Aziz R.F. (2020). Optimization of time and cost through learning curve analysis. Ain Shams Eng. J..

[75] Badakhshan N., Shahriar K., Afraei S., Bakhtavar E. (2023). Determining the environmental costs of mining projects: a comprehensive quantitative assessment. Resour. Policy.

[76] Brandl A., Bartsch K., Wilke J., Schleip R. (September, 2023).

[77] Nikjow M.A., Liang L., Qi X., Sepasgozar S.M.E., Chileshe N. (2021). Triggers of delays in international projects using engineering procurement and construction delivery methods in the belt and road initiative: case study of a high-speed railway projects. Sustain. Times.

[78] Damayanti A.A., Lanti Y., Dewi R. (2023). Validity and reliability of balanced nutrition knowledge and eating behavior questionnaires among adolescent girls.

[79] Smi I., Gra A., Kr K., Klaudia S., Strzelecki D. (October, 2023).

[80] Nawaz A., Chen J., Su X. (2023). Factors in critical management practices for construction projects waste predictors to C&DW minimization and maximization. J. King Saud Univ. Sci..

[81] Antoniou F. (2023).

[82] Edwards S.T., Peterson K., Chan B., Anderson J., Helfand M. (2017). “Effectiveness of intensive primary care interventions. A Systematic Review.

[83] Ahmed N., Abdel-hamid M., El-razik M.M.A., El-dash K.M. (2021). Impact of sustainable design in the construction sector on climate change. Ain Shams Eng. J..

[84] Antoniou F. (2021).

[85] Okudan O., Çevikba M. (2024).

[86] Zhong S., Elhegazy H., Elzarka H. (2022). Key factors affecting the decision-making process for buildings projects in Egypt. Ain Shams Eng. J..

[87] Ammar T., Abdel-monem M., El-dash K. (2022). Risk factors causing cost overruns in road networks. Ain Shams Eng. J..

[88] Gebrel M. (August, 2022).

[89] Álvarez-Pozo A.H., Parma-García M.I., Ortiz-Marcos I., Bautista L.F., Atanes-Sánchez E. (2024). Analysis of causes of delays and cost overruns as well as mitigation measures to improve profitability and sustainability in turnkey industrial projects. Sustain. Times.

