# Supplementary material for: A mixed-method investigation of the root causes of construction project delays in Afghanistan
Source: Heliyon. 2025 Jan 13;11(2):e41923. doi: 10.1016/j.heliyon.2025.e41923 (PMC11791142; doi:10.1016/j.heliyon.2025.e41923)
Supplement: Multimedia component 1 [file mmc1.pdf]

Cronbach's Alpha Test for Questionnaire Reliability

Introduction

Urban Cronbach's Alpha is a well-established coefficient in psychometrics and statistics used to assess the internal consistency reliability of a scale or test. In simpler terms, it evaluates how consistently a set of questions within a questionnaire measures a single underlying construct. The Cronbach's Alpha formula mathematically captures this concept:

$$\propto = \left(\frac{k}{k-1}\right)\left(1 - \frac{\sum_{i=1}^{k} \sigma^2 i}{\sigma^2 t}\right)$$

Here, α represents the Alpha coefficient (ranging from 0 to 1), k represents the number of questions in the questionnaire, σi represents the variance of each individual question, and σt represents the variance of the total score obtained by summing all the questions. Essentially, the formula calculates the ratio between the variance of the total scores and the sum of the variances of each item, adjusted for the number of items. A higher Alpha value indicates greater internal consistency, meaning the questions within the questionnaire are more likely to be measuring the same construct in a consistent manner.

Questionnaire Evaluation by Stakeholders:

The responses from the pilot survey participants provide valuable insights into the opinions of the four main stakeholders in the Afghan construction sector: project managers (PM), lecturers (Lect), contractors (Cont), and consultants (Cons). These stakeholders were asked to evaluate the importance of various aspects of construction projects using a five-point scale, ranging from 1 (very low importance) to 5 (very high importance). The tables below summarize the responses for Questionnaire.

Table 1: Responses to Questionnaire

Participants	Q1	Q2	Q3	Q4	Q5	Q6	Q7	Q8	Q9	Q10	Q11	Q12	Q13	Q14	Q15
PM	1	1	1	1	1	1	1	1	1	1	5	5	5	3	2
PM	3	5	3	3	3	3	2	2	2	1	5	5	4	4	4
PM	5	5	5	5	5	5	4	5	5	5	4	3	3	2	5
PM	5	5	5	5	5	5	5	5	5	5	3	4	3	3	4
PM	5	5	4	4	4	4	4	2	2	3	5	5	5	5	2
PM	1	1	1	1	1	1	1	1	1	1	4	4	1	1	5
PM	1	3	3	4	4	4	5	5	5	5	4	3	4	2	4
Lect	1	4	3	5	2	5	5	4	4	3	3	3	3	3	4
Lect	5	3	3	4	4	4	5	5	5	5	5	5	4	4	3
Lect	1	4	4	4	5	5	5	5	5	4	4	4	5	5	5
Lect	1	3	3	3	4	4	4	4	5	5	5	4	1	2	2
Lect	1	1	1	3	4	5	3	4	4	5	4	4	4	4	2

Participants	Q1	Q2	Q3	Q4	Q5	Q6	Q7	Q8	Q9	Q10	Q11	Q12	Q13	Q14	Q15
Cont	5	4	4	4	4	3	3	2	2	3	3	2	1	1	3
Cont	1	5	3	3	3	4	4	5	5	5	5	1	5	5	5
Cont	5	2	3	5	4	1	2	5	4	3	3	3	3	5	3
Cont	5	4	4	4	3	3	5	1	2	3	4	2	3	2	1
Cont	1	2	5	4	1	3	2	3	5	4	3	3	1	4	5
Cons	4	3	4	5	2	3	4	4	4	3	2	2	1	5	4
Cons	2	2	2	4	3	1	1	1	2	1	4	3	1	4	1
Cons	1	3	2	3	4	5	3	2	1	2	3	3	4	3	3

Note: PM = Project Manager, Lect = Lecturer, Cont = Contractor, Cons = Consultant

Calculation: Reliability Assessment (Cronbach's Alpha) for Questionnaire

Internal consistency, a measure of how well the items within a questionnaire assess a single construct, is crucial for reliable data collection. We employed Cronbach's Alpha to assess the internal consistency of Questionnaire.

$$\propto = \left(\frac{k}{k-1}\right)\left(1 - \frac{\sum_{i=1}^{k} \sigma^2 i}{\sigma^2 t}\right)$$

$$\propto = \left(\frac{15}{15-1}\right)\left(1 - \frac{30.2632}{139.568}\right)$$

$$\propto = 0.83911$$

Results:

The Cronbach's Alpha coefficient for Questionnaire is $\propto = 0.83911$. Commonly used benchmarks for interpreting Cronbach's Alpha are:

- 0.7 or higher: Acceptable reliability
- 0.8 or higher: Good reliability
- 0.9 or higher: Excellent reliability

Interpretation:

Based on the Cronbach's Alpha coefficients of 0.83911 Questionnaire demonstrate good internal consistency reliability. This suggests that the items within questionnaire are measuring a single underlying construct relatively consistently. The Cronbach's Alpha analysis indicates that questionnaire possess good internal consistency reliability. This finding supports the use of the questionnaire to achieve the objectives of the research.
