# Supplementary material for: A mixed-method investigation of the root causes of construction project delays in Afghanistan
Source: Heliyon. 2025 Jan 13;11(2):e41923. doi: 10.1016/j.heliyon.2025.e41923 (PMC11791142; doi:10.1016/j.heliyon.2025.e41923)
Supplement: Multimedia component 2 [file mmc2.pdf]

Multicollinearity Test for Data Reliability

This appendix presents the results of the multicollinearity test conducted to assess potential issues of multicollinearity among the independent variables used in the quantitative analysis of construction project delays in Afghanistan. Multicollinearity occurs when two or more independent variables are highly correlated, potentially leading to inflated standard errors and unreliable regression coefficients.

This research employed a questionnaires survey for data collection.

- **Questionnaire:** This questionnaire focuses on identifying the ranking of delay causes based on stakeholder perspectives. It targets five main stakeholder groups involved in Afghan construction projects: Government, Consultant, Contractor, Project manager and Lecturer. All delay causes were categorized in four main causative groups. Each group has a specific set of factors associated with potential delay causes.

Understanding the Statistical Tests

This section provides a brief explanation of the key statistical tests employed in the research:

- **Descriptive Statistics (Mean and Standard Deviation):** These measures summarize the data by providing the average value (mean) and the spread of the data points around the mean (standard deviation). They offer insights into the central tendency and variability of the data for various delay cause factors.
- **Skewness and Kurtosis:** These tests assess the symmetry (skewness) and "tailedness" (kurtosis) of the data distribution. Values within a specific range (generally between -1 and +1) indicate an approximately normal distribution, which is desirable for some statistical tests like regression analysis. Significant deviations from these ranges suggest a skewed or non-normal distribution, which might be addressed through data transformation techniques if necessary.
- **Variance Inflation Factor (VIF):** VIF helps identify multicollinearity among independent variables in regression analysis. High VIF values (generally above 10) suggest variables are highly correlated, potentially affecting the stability and interpretation of the regression coefficients. The multicollinearity test results presented in this appendix will utilize VIF to assess potential issues and determine if corrective actions are necessary.

- **Cronbach's Alpha (α):** This test evaluates the internal consistency of a questionnaire or scale. A high Cronbach's Alpha value (generally 0.70 or higher) indicates that the items within a questionnaire measure the same underlying concept reliably. The results presented in Appendix #6 detail the Cronbach's Alpha scores for questionnaire.

By understanding these statistical tests, you can better interpret the results presented in the data analysis chapters, particularly the findings related to the multicollinearity test. Following Table 1 is the details of the test for questionnaire.

Table 1. Multicollinearity Test Results for Questionnaire (Stakeholder Groups)

Groups	Items	Mean	Std. Dev	Skewness	Kurtosis	VIF	Cronbach α
Government	Gove1	4.22	.927	-1.317	1.637	1.960	0.846
	Gove2	3.74	1.065	-.586	-.212	2.176	
	Gove3	3.86	1.135	-.686	-.466	2.362	
	Gove4	3.62	1.259	-.605	-.690	2.645	
	Gove5	3.80	1.129	-.948	.260	2.972	
	Gove6	3.82	1.255	-1.129	.391	2.901	
	Gove7	3.66	1.265	-.770	-.406	2.310	
	Gove8	3.64	1.199	-.588	-.639	2.848	
	Gove9	3.91	1.116	-.812	-.202	2.835	
	Gove10	3.83	1.097	-.951	.452	2.778	
	Gove11	3.88	.954	-.952	1.232	2.808	
	Gove12	3.85	.984	-.868	.623	2.728	
	Gove13	3.89	.868	-.571	.100	2.571	
	Gove14	4.02	.919	-.962	.785	2.843	
	Gove15	4.14	.929	-1.138	1.314	2.809	
	Gove16	4.01	.971	-.921	.664	2.168	
	Gove17	4.00	1.076	-1.103	.652	2.756	
	Gove18	3.99	1.051	-1.092	.574	3.540	
Consultant	Cons1	4.10	1.027	-1.214	1.076	1.848	0.804
	Cons2	3.73	1.029	-.550	-.119	2.498	
	Cons3	3.75	1.159	-.573	-.610	2.164	
	Cons4	3.32	1.263	-.270	-.936	1.920	
	Cons5	3.66	1.219	-.796	-.232	2.922	
	Cons6	3.53	1.306	-.807	-.364	2.443	
	Cons7	3.68	1.329	-.794	-.544	3.000	
	Cons8	3.56	1.285	-.531	-.892	3.392	
	Cons9	3.66	1.222	-.514	-.793	2.836	
	Cons10	3.61	1.189	-.742	-.173	3.453	
	Cons11	3.71	1.066	-.839	.519	2.475	
	Cons12	3.74	1.145	-.759	-.195	2.513	
	Cons13	3.74	1.057	-.811	.331	2.293	
	Cons14	3.81	1.057	-.892	.359	2.371	
	Cons15	3.75	1.127	-.902	.262	2.720	
	Cons16	3.77	1.112	-.823	.036	3.162	
	Cons17	3.71	1.212	-.784	-.333	3.238	
	Cons18	4.00	1.114	-1.019	.106	2.362	

Groups	Items	Mean	Std. Dev	Skewness	Kurtosis	VIF	Cronbach α
	Cons19	3.88	1.199	-.889	-.156	2.732	
	Cons20	3.93	1.011	-.749	.106	2.255	
	Cons21	3.79	1.028	-.566	-.311	2.539	
	Cons22	3.83	1.105	-.822	-.112	3.676	
	Cons23	3.71	1.166	-.621	-.581	3.028	
	Cons24	4.11	.937	-1.138	1.164	2.410	
Contractor	Cont1	4.12	.935	-1.596	3.093	4.966	0.874
	Cont2	4.19	.886	-1.533	3.157	4.931	
	Cont3	4.22	.869	-1.443	2.589	4.921	
	Cont4	4.28	.771	-1.368	3.075	3.412	
	Cont5	4.24	.836	-1.476	3.084	3.842	
	Cont6	4.26	.824	-1.283	2.114	3.441	
	Cont7	4.18	.814	-1.061	1.426	4.522	
	Cont8	4.21	.782	-1.159	2.046	3.417	
	Cont9	4.23	.784	-1.416	3.414	2.305	
	Cont10	4.12	.797	-1.200	2.578	2.100	
	Cont11	4.07	.848	-1.345	2.727	2.300	
	Cont12	4.07	.850	-1.258	2.289	1.933	
	Cont13	4.05	.916	-1.261	1.897	2.037	
	Cont14	4.11	.909	-1.325	2.027	2.107	
	Cont15	4.19	.775	-1.093	1.930	1.740	
	Cont16	4.14	.802	-1.096	1.948	1.594	
	Cont17	4.12	.794	-.956	1.620	2.515	
	Cont18	4.10	.816	-.989	1.506	2.846	
	Cont19	4.16	.822	-.976	1.109	2.700	
	Cont20	4.00	.978	-1.025	.929	2.234	
	Cont21	3.90	.998	-.836	.419	2.065	
	Cont22	4.10	.843	-.907	1.197	2.313	
	Cont23	4.01	.849	-.835	.884	2.184	
	Cont24	3.86	1.050	-.935	.445	2.372	
	Cont25	3.80	1.170	-.936	.202	1.829	
	Cont26	3.76	1.150	-.832	.021	2.622	
	Cont27	3.90	1.063	-.990	.547	2.100	
	Cont28	3.71	1.218	-.835	-.238	2.015	
	Cont29	3.84	1.134	-1.052	.569	2.396	
	Cont30	3.84	1.209	-1.122	.483	2.779	
	Cont31	3.81	1.225	-.999	.048	2.552	
	Cont32	3.52	1.331	-.535	-.960	2.704	
	Cont33	3.82	1.154	-.780	-.200	2.665	
	Cont34	3.62	1.205	-.804	-.146	2.284	
	Cont35	4.07	.980	-1.266	1.657	1.789	
	Cont36	4.05	.959	-1.003	.710	1.713	
	Cont37	3.92	.943	-1.107	1.536	1.942	
	Cont38	3.98	.914	-.930	.890	2.055	
	Cont39	3.95	.909	-.716	.363	2.074	
	Cont40	4.22	.927	-1.317	1.637	1.960	
Security	Secu1	3.74	1.065	-.586	-.212	2.176	0.742
	Secu2	3.86	1.135	-.686	-.466	2.362	
	Secu3	3.62	1.259	-.605	-.690	2.645	
	Secu4	3.80	1.129	-.948	.260	2.972	
	Secu5	3.82	1.255	-1.129	.391	2.901	
	Secu6	3.66	1.265	-.770	-.406	2.310	

Groups	Items	Mean	Std. Dev	Skewness	Kurtosis	VIF	Cronbach α
	Secu7	3.64	1.199	-.588	-.639	2.848	
	Secu8	3.91	1.116	-.812	-.202	2.835	
	Secu9	3.83	1.097	-.951	.452	2.778	
	Secu10	3.88	.954	-.952	1.232	2.808	
	Secu11	3.85	.984	-.868	.623	2.728	
	Secu12	3.89	.868	-.571	.100	2.571	
	Secu13	4.02	.919	-.962	.785	2.843	
	Secu14	4.14	.929	-1.138	1.314	2.809	
	Secu15	4.01	.971	-.921	.664	2.168	

Note: Gove = Government, Cons = Consultant, Cont = Contractor, Secu = Security.
